# TopBP1 Governs Hematopoietic Stem/Progenitor Cells Survival in Zebrafish Definitive Hematopoiesis

**DOI:** 10.1371/journal.pgen.1005346

**Published:** 2015-07-01

**Authors:** Lei Gao, Dantong Li, Ke Ma, Wenjuan Zhang, Tao Xu, Cong Fu, Changbin Jing, Xiaoe Jia, Shuang Wu, Xin Sun, Mei Dong, Min Deng, Yi Chen, Wenge Zhu, Jinrong Peng, Fengyi Wan, Yi Zhou, Leonard I. Zon, Weijun Pan

**Affiliations:** 1 Key Laboratory of Stem Cell Biology, Institute of Health Sciences, Shanghai Institutes for Biological Sciences, Chinese Academy of Sciences & Shanghai Jiao Tong University School of Medicine, Shanghai, China; 2 Department of Biochemistry and Molecular Biology, Bloomberg School of Public Health, Johns Hopkins University, Baltimore, Maryland, United States of America; 3 State Key Laboratory for Medical Genomics, Shanghai Institute of Hematology, RuiJin Hospital, Shanghai Jiao Tong University School of Medicine, Shanghai, China; 4 Department of Biochemistry and Molecular Biology, The George Washington University Medical School, Washington, D.C., United States of America; 5 Key Laboratory for Molecular Animal Nutrition, Ministry of Education, College of Animal Sciences, Zhejiang University, Hangzhou, China; 6 Department of Oncology and The Sidney Kimmel Comprehensive Cancer Center, Johns Hopkins Medical Institutions, Baltimore, Maryland, United States of America; 7 Stem Cell Program, Hematology/Oncology Program at Children's Hospital Boston and Dana Farber Cancer Institute, Harvard Medical School, Boston, Massachusetts, United States of America; Stanford University School of Medicine, UNITED STATES

## Abstract

In vertebrate definitive hematopoiesis, nascent hematopoietic stem/progenitor cells (HSPCs) migrate to and reside in proliferative hematopoietic microenvironment for transitory expansion. In this process, well-established DNA damage response pathways are vital to resolve the replication stress, which is deleterious for genome stability and cell survival. However, the detailed mechanism on the response and repair of the replication stress-induced DNA damage during hematopoietic progenitor expansion remains elusive. Here we report that a novel zebrafish mutant^cas003^ with nonsense mutation in *topbp1* gene encoding topoisomerase II β binding protein 1 (TopBP1) exhibits severe definitive hematopoiesis failure. Homozygous *topbp1^cas003^* mutants manifest reduced number of HSPCs during definitive hematopoietic cell expansion, without affecting the formation and migration of HSPCs. Moreover, HSPCs in the caudal hematopoietic tissue (an equivalent of the fetal liver in mammals) in *topbp1^cas003^* mutant embryos are more sensitive to hydroxyurea (HU) treatment. Mechanistically, subcellular mislocalization of TopBP1*^cas003^* protein results in ATR/Chk1 activation failure and DNA damage accumulation in HSPCs, and eventually induces the p53-dependent apoptosis of HSPCs. Collectively, this study demonstrates a novel and vital role of TopBP1 in the maintenance of HSPCs genome integrity and survival during hematopoietic progenitor expansion.

## Author Summary

The rapidly proliferating hematopoietic stem/progenitor cells (HSPCs) require well-established DNA damage response/repair pathways to resolve the DNA replication stress-induced DNA damage, which is deleterious for the genome stability and cell survival. Impairment of these pathways could lead to the progressive bone marrow failure (BMF) and hematopoietic malignancies. Here we reported a novel function of topoisomerase II β binding protein 1 (TopBP1) in definitive hematopoiesis through characterizing zebrafish mutant^*cas003*^ with a nonsense mutation in *topbp1* gene encoding TopBP1. The homozygous *topbp1* mutants manifested decreased HSPCs during their pool expansion in the caudal hematopoietic tissue (CHT, an equivalent of the fetal liver in mammals) due to the p53-dependent apoptosis. Further investigation revealed that the deficient TopBP1-ATR-Chk1 pathway upon DNA replication stress in *topbp1* mutants led to accumulated DNA damage and further affected HSPCs survival. These studies therefore emphasized the importance of *topbp1* function as well as DNA damage response pathways during the fetal HSPC rapid proliferation.

## Introduction

Hematopoietic stem/progenitor cells (HSPCs) possess the capabilities of self-renewal and differentiation into all lineages of mature blood cells [1]. Dysregulated self-renewal of HSPCs is tightly associated with the human blood diseases including leukemia and bone marrow failure (BMF) syndrome [2–4]. Previous studies have illustrated that the genes causative for adult hematopoietic diseases virtually play critical roles in the early hematopoiesis [5,6]. Therefore, exploring the unknown genetic regulators of HSPCs in the hematopoiesis would give us better understanding of the sophisticated mechanisms of hematopoietic diseases in adults.

Recently, zebrafish has emerged as an excellent animal model to study the development of hematopoiesis [7–9]. With multiple unique advantages including external fertilization and development, optically transparent embryos, small size and high fecundity, zebrafish is extraordinarily suitable for the unbiased large scale forward genetics screening to identify novel genes regulating HSPCs self-renewal in the embryonic development [10]. More importantly, the hematopoietic anatomy and the critical transcriptional factors involved in the development of hematopoiesis are highly conserved between zebrafish and mammals [1,11]. Similar to mammals, zebrafish hematopoiesis consists of two waves of hematopoiesis, *i*.*e*. primitive hematopoiesis and definitive hematopoiesis. The primitive hematopoiesis takes place in the anterior lateral plate mesoderm (ALPM) and intermediate cell mass (ICM) at ~12–14 somites stage, producing primitive macrophages and erythrocytes, respectively [12]. In zebrafish definitive hematopoiesis, HSPCs originate in the ventral wall of dorsal aorta (an equivalent of the aorta-gonad-mesonephros [AGM] in mammals) through endothelium to hematopoietic transition (EHT) from 26 hours post fertilization (hpf) [13,14], and then colonize in caudal hematopoietic tissue (CHT, an equivalent to the fetal liver [FL] in mammal) (at 2 days post fertilization [dpf]), thymus (at 3dpf) and ultimately kidney marrow to support adult hematopoiesis (equivalent to bone marrow (BM) in mammal) (after 5dpf) [15,16]. During fetal hematopoiesis in CHT, the nascent HSPCs undergo extensive proliferation for the pool expansion to support the embryo development [15]. It has been reported that 95–100% of HSPCs are actively cycling in the mouse fetal liver, whereas most of adult HSPCs are in a quiescent state [17].

During DNA replication, the slowed or stalled DNA replication fork, which is termed as DNA replication stress, occurs frequently due to intracellular and extracellular sources including the by-products of cellular metabolism (*e*.*g*. dNTP misincorporation, reactive oxygen species *etc*.), ultraviolet light and chemical mutagens [18,19]. Because the stalled replication forks are vulnerable and the collapse of the forks can result in DNA double strand breaks (DSBs) that are deleterious for the genome stability and cell survival, the DNA replication stress-induced DNA damage needs to be efficiently resolved by DNA damage response (DDR) pathways [18]. The phosphoinositide kinase-related kinase ataxia telangiectasia mutated (ATM) and ATM and Rad3-related (ATR) are two important kinases involved in DDR. ATM mainly participates in the DSBs response, whereas ATR is activated by the single-stranded DNA (ssDNA) damage and DNA replication stress [20]. Recent studies have shed the light on the association between hematopoietic homeostasis and DDR. DDR impairment can lead to progressive BMF and hematopoietic malignancies [21–23]. Fanconi anemia (FA) pathway, which consists of 15 FA genes, mainly participates in repairing the DNA interstrand crosslinks (ICL). Most of the FA genes are associated with the replication fork protection and ATR activation pathway [24,25], and they are causally mutated in BMF or acute myelogenous leukemia [26].

Topoisomerase II β binding protein 1 (TopBP1) is a structurally and functionally conserved protein from yeast to human, which is essential as a scaffold protein in DNA replication initiation and DNA damage checkpoint activation [27–30]. TopBP1 plays a vital role in the DDR, it mainly protects against the ssDNA damage and DNA replication stress through the TopBP1-ATR-Chk1 axis [31–33]. In this process, the stalled replication forks will generate a typical double-stranded DNA-single-stranded DNA (dsDNA-ssDNA) structure. Following the replication protein A (RPA) coating, TopBP1-associated proteins including Rad9-Rad1-Hus1 (9-1-1 complex), ATR interaction protein (ATRIP) and ATR are recruited to the damage locus, then TopBP1 largely activates the ATR kinase activity through its ATR activation domain (AAD), which triggers the phosphorylation of Chk1 and stabilization of replication forks until the stress is resolved [34–38]. Other TopBP1 interacting components also facilitate the establishment of the TopBP1-ATR-Chk1 axis, including the mediator of DNA-damage checkpoint 1 (MDC1) and BRCA1 interacting protein C-terminal helicase (BRIP1, aka, FANCJ) [39–42].

Although the cellular function of TopBP1 has been established, its physiological role, especially the tissue specific requirement, is still largely unknown. TopBP1 null mice are embryonic lethal due to accumulated DNA damage and reduced cell proliferation, which is phenocopied by TopBP1 ^W1147R^ knock-in mice with abrogated AAD domain of TopBP1 [43,44]. Moreover, neuronal specific deletion of TopBP1 in mice demonstrates that TopBP1 is essential for neural progenitor cells to survive from the DNA replication stress [45]. Specific disruption of TopBP1 in the lymphoid cells blocks lymphocyte development due to aberrant V(D)J rearrangement [46]. However, whether TopBP1 participates in the HSPCs development is still unknown.

Here we report a novel zebrafish mutant^*cas003*^, in which HSPCs can be generated normally, but fail thereafter in definitive hematopoiesis. Positional cloning and functional validation indicated that a nonsense mutation-caused C-terminal truncation of TopBP1 was responsible for its subcellular mislocalization and hematopoietic deficits. Disrupted TopBP1-ATR-Chk1 pathway and the accumulation of DNA damage were associated with the HSPCs defect and triggered apoptosis via a p53-dependent pathway. Our findings demonstrate that *topbp1* is essential for the HSPCs survival under extensive DNA replication stress during the highly proliferative fetal definitive hematopoiesis.

## Results

### Mutants^*cas003*^ display defective definitive hematopoiesis

**Fig 1:**
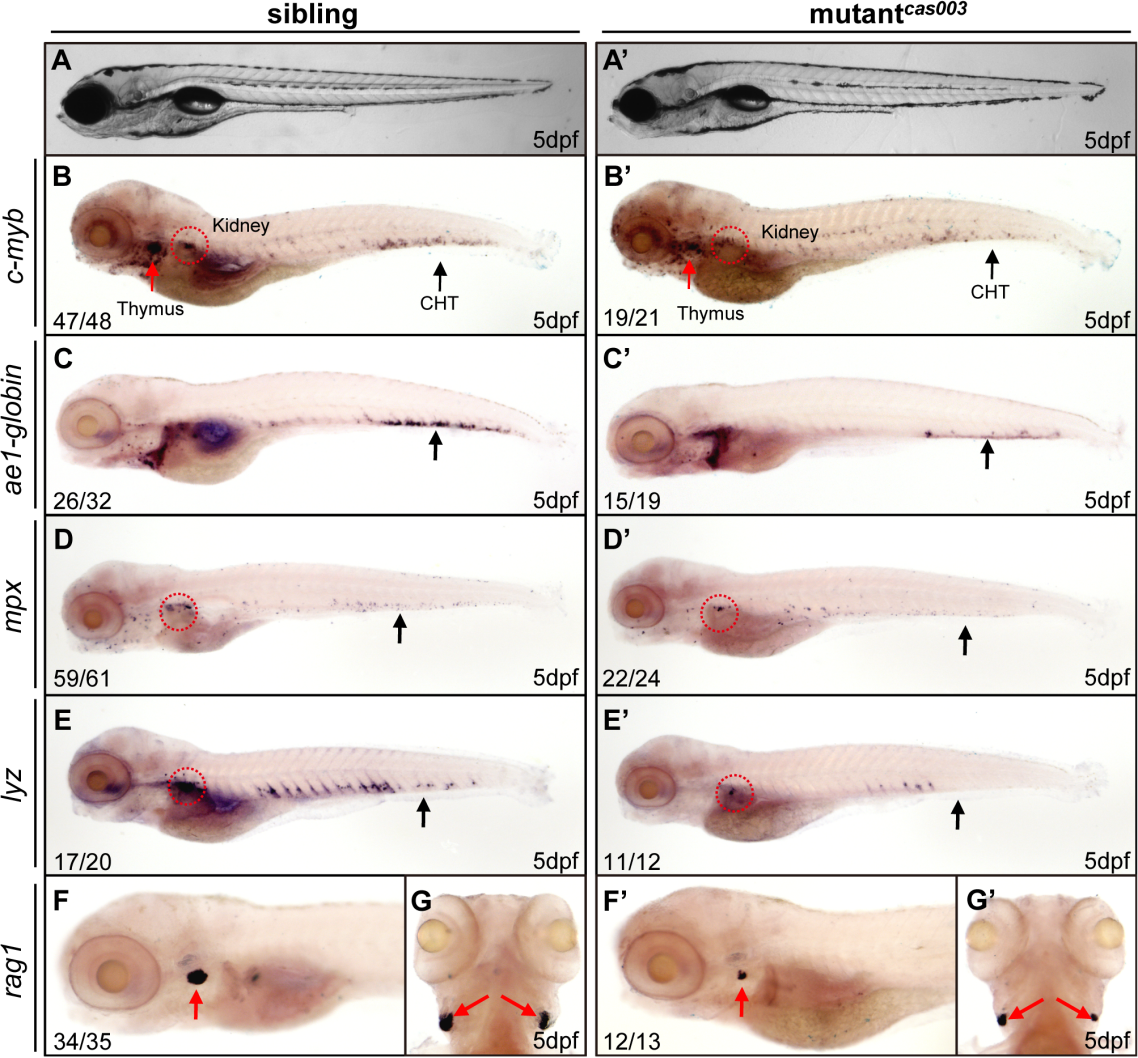
The definitive hematopoiesis is defective in zebrafish mutant^*cas003*^ embryos. (A, A’) The bright field images of zebrafish wild-type sibling (A) and mutant^***cas003***^ embryos (A’) showing no obvious difference at 5dpf. (B-G’) Whole-mount *in situ* hybridization (WISH) results of *c-myb*, *ae1-globin*, *mpx*, *lyz* and *rag1* showing defective definitive hematopoiesis in mutant^***cas003***^ embryos (B’-G’) but not in sibling embryos (B-G) at 5dpf. The penetrance of the indicated phenotype is shown in the bottom left of each panel. (B-F, B’-F’) Lateral views; (G, G’) dorsal views. Black arrows indicate the position of caudal hematopoietic tissue (CHT); red arrows and circles show the position of thymus and kidney, respectively.

To explore new genes and regulatory mechanisms in vertebrate definitive hematopoiesis, we carried out a large-scale forward genetics screen on ENU-mutagenized F2 families in zebrafish by whole mount *in situ* hybridization (WISH) using *c-myb* probe (a key transcription factor and marker of HSPCs) [15,47]. In 5dpf wild-type zebrafish embryos, *c-myb* was expressed in all hematopoietic tissues including caudal hematopoietic tissue (CHT), thymus, and kidney (Fig 1); whereas homozygous mutants^*cas003*^ displayed normal morphogenesis (Fig 1A–A’), but dramatically decreased *c-myb* expression in CHT, kidney and thymus (Fig 1B–B’), suggesting the expansion of HSPCs was defective. To confirm the defective definitive hematopoiesis in mutants^*cas003*^, we further examined the expression of downstream hematopoietic lineage cell markers including *ae1-globin* (erythrocyte marker), *mpx* (granulocyte marker), *lyz* (macrophage marker) and *rag1* (lymphocyte marker). The expression of all these markers was substantially decreased in the homozygous mutant^*cas003*^ embryos at 5dpf (Fig 1C–G’), which suggested hematopoiesis failure.

Recent studies have demonstrated that vasculogenesis and blood flow are essential for HSPCs initiation and maintenance [48,49]. We examined the expression pattern of a pan-endothelial cell marker *flk1* at 36hpf and an artery vessel marker *ephrinB2* at 26hpf respectively, our results revealed that both of them were intact in mutant^*cas003*^ (S1A–S1D Fig). Consistently, heart beating rate and blood circulation were comparable between mutant^*cas003*^ and sibling control (S1 and S2 movie). In addition, live observation on mutant^*cas003*^, within Tg(*fli1*: EGFP) transgenic background [50], indicated that the vascular plexus in the CHT region was normal from 2dpf to 5dpf (S1E–S1L Fig). We further investigated the primitive hematopoiesis in mutant^*cas003*^. The WISH analysis data demonstrated that the expression of primitive hematopoietic cell markers were identical between siblings and mutant^*cas003*^ at 22hpf, including *scl* (hematopoietic progenitor marker), *gata1* (erythrocyte progenitor marker), *pu*.*1* (myeloid progenitor marker), *lyz*, *l-plastin* (myeloid cell marker) and *mpx* (S2A–S2L Fig, quantified in M). Taken together, we concluded that mutant^*cas003*^ displayed specific deficiency in definitive hematopoiesis during zebrafish circulation system development.

### HSPCs defects initiate in the CHT of mutant^*cas003*^

**Fig 2:**
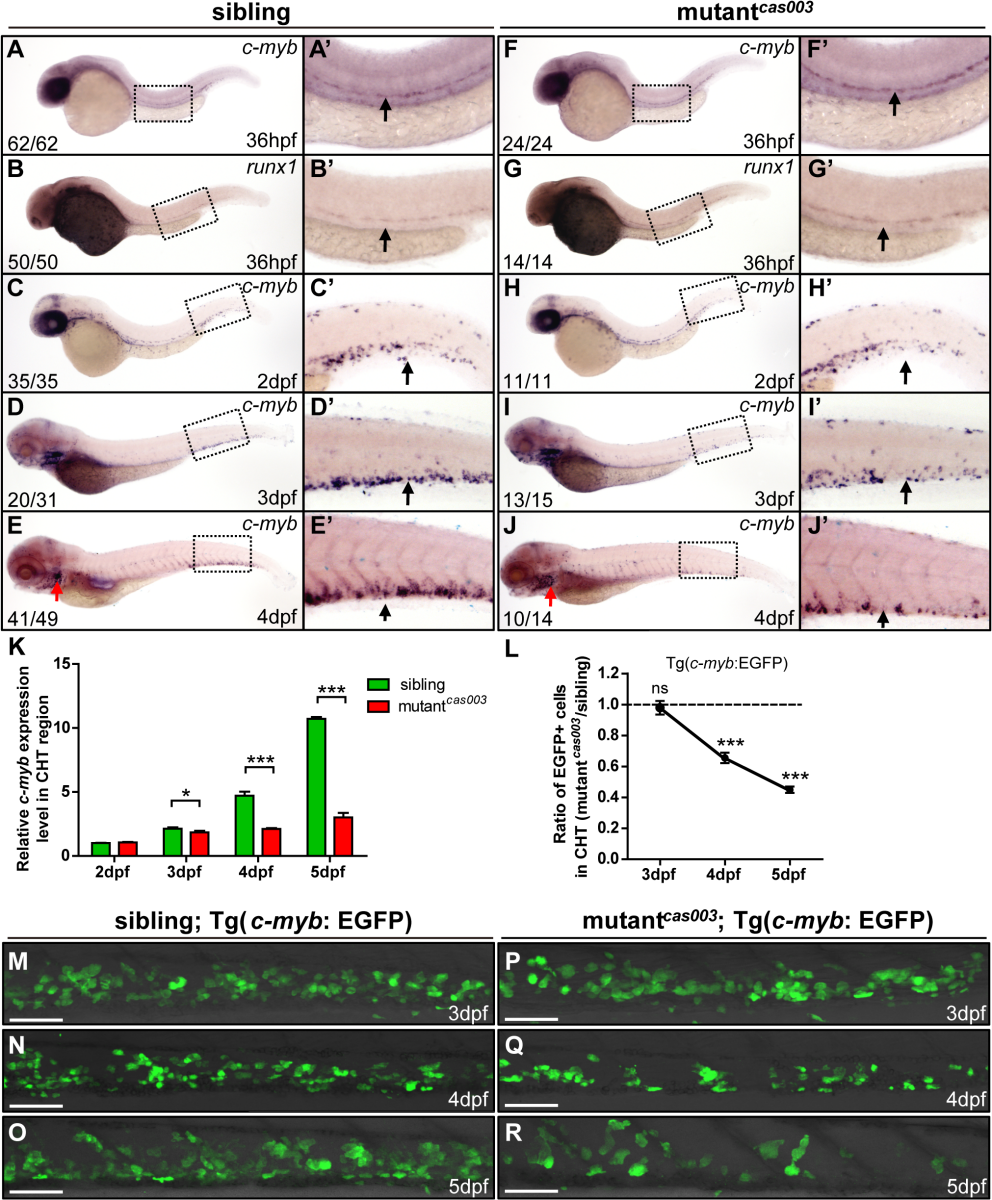
The number of HSPCs is reduced in the mutant^*cas003*^ CHT. (A-J’) The time-course analysis of definitive HSPCs from 36hpf to 4dpf by WISH with *c-myb* (A, A’, C-E’, F, F’, H-J’) or *runx1* (B, B’, G, G’) indicating the HSPCs are decreased from 3dpf in mutant^***cas003***^ embryos comparing to siblings. The penetrance of the indicated phenotype is shown in the bottom left of each panel. (A’-J’) Enlarged views of AGM or CHT regions indicated by the dotted rectangles in the left columns. Black arrows represent the AGM (A’, B’, F’, G’) or CHT (C’-E’, H’-J’); red arrows indicate the thymus. (K) Quantitative PCR analysis of *c-myb* expression level in the tail region from 2dpf to 5dpf showing the attenuated *c-myb* expression after 3dpf in mutant^***cas003***^ embryos comparing to siblings. Error bars represent SEM. *, *p*<0.05; ***, *p*<0.001. (L-R) Immunofluorescence stain of EGFP in the CHT region in mutant^***cas003***^; Tg(*c-myb*: EGFP) embryos (P-R) and siblings (M-O) from 3dpf to 5dpf. The statistics result of the ratio of EGFP^***+***^ cell number in the CHT region of mutant^***cas003***^ embryos to that in the siblings is indicated in L (n>6 embryos are counted in each panel). These results indicate that EGFP^***+***^ HSPCs begin to be decreased obviously in CHT region in mutant^***cas003***^ embryos comparing to siblings from 4dpf. Scale bars represent 50μm. Error bars represent SEM; ns, no significance; *** represents *p*<0.001.

HSPCs are generated from the ventral wall of dorsal aorta through the endothelia to hematopoietic transition (EHT) from 26hpf [13,14], and then migrate to the CHT, a proliferative hematopoietic microenvironment, for pool expansion at 2dpf [15,16]. To figure out when the HSPCs defect initiated in mutant^*cas003*^, we performed a time course analysis of *c-myb* expression from 36hpf to 5dpf. The WISH results demonstrated that the generation of HSC was intact in mutant^*cas003*^ as both *c-myb* and *runx1* [51] expression were undisturbed at 36hpf (Fig 2A–B’ and 2F–G’), and the *c-myb* expression was still intact in the CHT at 2dpf in mutant^*cas003*^ (Fig 2C–C’ and 2H–H’). However, mutant^*cas003*^ displayed reduced *c-myb* expression in the CHT at 3dpf (Fig 2D–D’ and 2I–I’), and such defect was more profound at 4dpf (Fig 2E–E’ and 2J–J’), indicating that the HSPCs proliferation or maintenance was impaired in the CHT of mutant^*cas003*^. To consolidate this discovery, we carried out quantitative RT-PCR analysis on the *c-myb* mRNA level in zebrafish tails region including CHT from 2dpf to 5dpf. As expected, the *c-myb* expression level was attenuated from 3dpf to 5dpf (Fig 2K), which was consistent with the results of WISH analysis. To further confirm these findings, we crossed mutant^*cas003*^ with Tg(*c-myb*: EGFP), in which HSPCs could be visualized by EGFP [52]. Statistically significant reduction of EGPF^+^ cells was observed at 4dpf (Fig 2L, 2N and 2Q) and was more severe at 5dpf in mutant^*cas003*^ (Fig 2L, 2O and 2R) (Due to the long half-life of EGFP protein, the dynamics of *c-myb* expression indicated via Tg(*c-myb*: EGFP) was delayed, compared to WISH analysis via *c-myb* probe [5]). Collectively, our data revealed that, in mutant^*cas003*^, neither HSPCs specification in AGM nor their migration to CHT was affected, but their transitory expansion in the CHT was compromised.

### The *topbp1* gene is disrupted in mutant^*cas003*^

**Fig 3:**
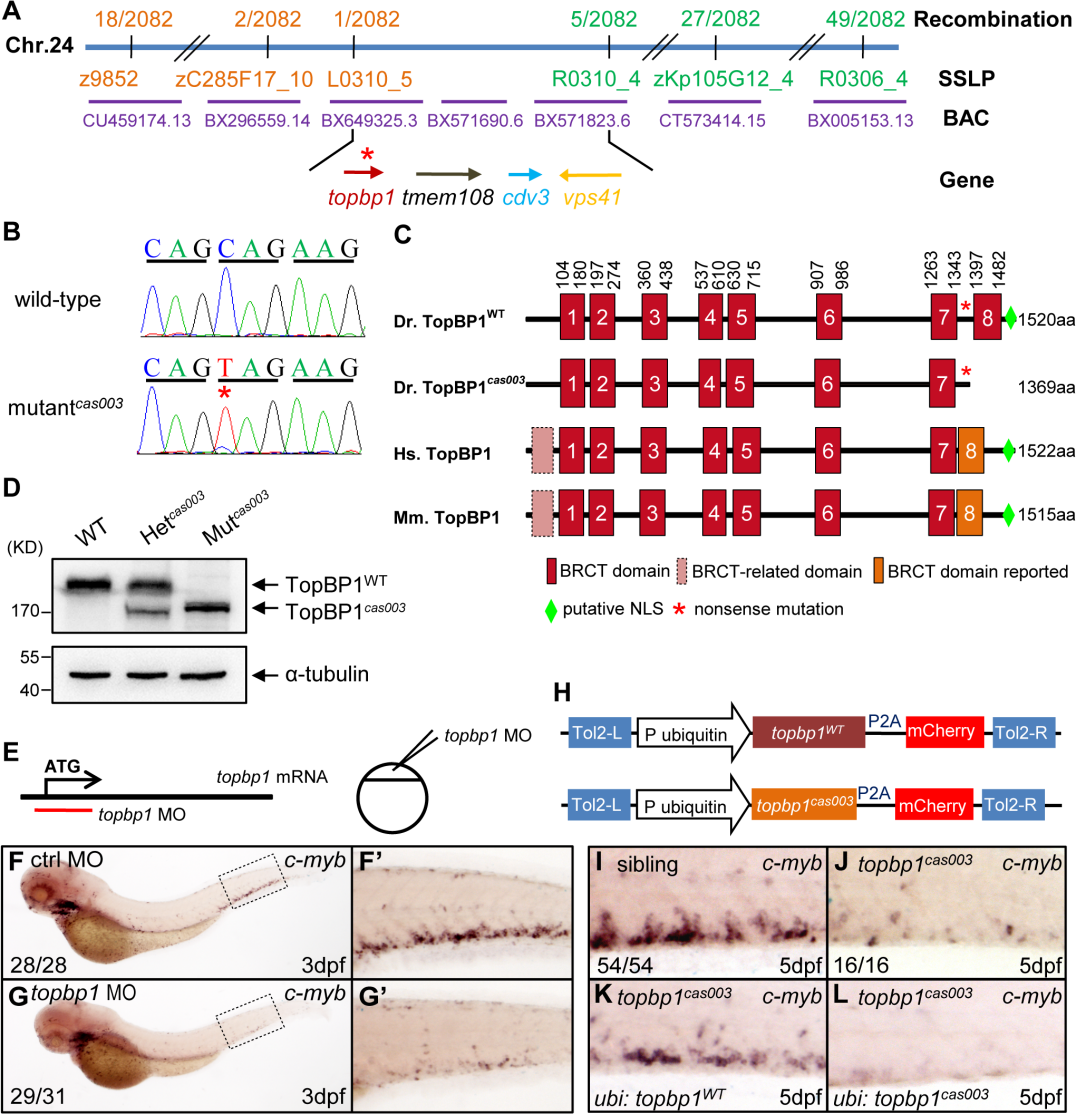
The *topbp1* gene is disrupted in mutant^*cas003*^. (A) Positional cloning of mutant^***cas003***^. Bulk segregation analysis (BSA) revealed that the mutation occurred on chromosome 24 (Chr. 24). After high-resolution mapping with SSLPs, the point mutation was flanked by SSLP markers L0310_5 (1 recombinant out of 2082 meiosis) and R0310_4 (5 recombinants out of 2082 meiosis). This region contains four genes including *topbp1*, *tmem108*, *cdv3* and *vps41*. The blue line represents Chr. 24; the positions and the recombinations of the SSLP markers on Chr. 24 or BACs are indicated. The SSLP markers which are on the same side of the mutation site are shown in the same color. (B) The coding region of *topbp1* was sequenced in the wild-type sibling (top) and mutant^***cas003***^ (bottom). There is a C to T mutation in the mutant^***cas003***^ embryos, which is a nonsense mutation. (C) Comparison of vertebrate TopBP1 and zebrafish TopBP1^***cas003***^. As human TopBP1 (Hs. TopBP1) and mice TopBP1 (Mm. TopBP1), zebrafish TopBP1^***WT***^ (Dr. TopBP1) contains eight BRCT domains, while the eighth BRCT domain and putative nuclear localization signal (NLS) is missing in TopBP1^***cas003***^ due to the nonsense mutation (*). The red and pink BRCT domains were predicted by SMART software. The orange BRCT domains were reported previously. The molecular sizes of the protein are indicated in the right side. (D) Western blotting analysis showing reduced protein size of TopBP1^***cas003***^. (E-G’) *Topbp1* morphants can phenocopy *topbp1* ^***cas003***^. (E) *Topbp1* MO can block the translation of *topbp1* mRNA. The *topbp1* MO, as validated in S3 Fig, was injected into one-cell stage wild-type embryos to produce *topbp1* morphants. (F-G’) WISH results of *c-myb* in the control morphants (F, F’) and *topbp1* morphants (G, G’) at 3dpf. *topbp1* morphants show decreased *c-myb* expression. (F’, G’) The enlarged views of the dotted rectangle region in the left columns. The penetrance of the indicated phenotype is shown in the bottom left of each panel. (H) Construction of the plasmids used in Tol2-transposease-mediated rescue assays. *topbp1* ^***WT***^ and *topbp1* ^***cas003***^ driven by the ubiquitin promoter, followed by the P2A peptide and the mCherry coding sequence, are cloned into Tol2 transposon vector. These constructs are abbreviated as *ubi*: *topbp1* ^***WT***^ and *ubi*: *topbp1* ^***cas003***^ respectively. (I-L) WISH analysis with *c-myb* probe in the CHT region (at 5dpf) of sibling, *topbp1* ^***cas003***^ mutant and *topbp1* ^***cas003***^ mutant with transient transgenesis of *ubi*: *topbp1* ^***WT***^ or *ubi*: *topbp1* ^***cas003***^. 28 embryos out of 45 *topbp1* ^***cas003***^ mutants were rescued by *topbp1* ^***WT***^, but none by *topbp1* ^***cas003***^ (n = 14).

In order to elucidate the mechanism of hematopoietic failure in mutant^*cas003*^, we carried out positional cloning of the mutant. The mutation was first mapped to chromosome 24 by bulk segregation analysis (BSA). With a high resolution mapping approach, the mutation was revealed to be flanked by two closely linked SSLP markers, L0310_5 and R0310_4. The flanked region contained four candidate genes: *topbp1* (topoisomerase II β binding protein 1), *tmem108*, *cdv3* and *vps41* (Fig 3A). After sequencing cDNA of all 4 genes, we identified a C to T nonsense mutation in *topbp1* gene in mutant^*cas003*^ (Fig 3B), and confirmed this result through genomic sequencing. This mutation caused an earlier stop codon before the eighth BRCT (BRCA1 C-terminus) domain and a putative C-terminus nuclear localization signal (NLS) of TopBP1 protein (Fig 3C). This truncated form of endogenous TopBP1 (TopBP1^*cas003*^) protein was further confirmed by immunoblotting analysis of the CHT of heterozygote (Het ^*cas003*^) and mutant^*cas003*^ embryos at 3dpf (Fig 3D).

In order to examine whether the disruption of *topbp1* was causative for phenotype of mutant^*cas003*^, we injected a validated *topbp1* ATG morpholino oligo (MO) (S3A–S3B Fig) into one-cell stage wild-type embryos to block the translation of endogenous *topbp1* mRNA (Fig 3E). Since *topbp1* MO acted in a dose-dependent manner (S3C Fig), we applied morpholino microinjection causing no morphologic phenotype in the following studies. *Topbp1* morphants manifested severe defective definitive hematopoiesis as that in mutants^*cas003*^ from 36hpf to 5dpf, while the primitive hematopoiesis at 22hpf, HSPC generation in AGM at 36hpf and vascular system in CHT at 3dpf were all intact in the morphants (Fig 3F–G’ and S4A–S4R Fig). To further consolidate our findings, we performed rescue assay by ectopic expression of wild-type *topbp1* in mutant^*cas003*^. Consistent with previous report on the instability of *topbp1* mRNA [53], ectopic expression of TopBP1 was barely detected at 3dpf after injection of *in vitro* synthesized *topbp1* mRNA into 1-cell stage embryos. In order to overcome this obstacle, we employed a Tol2 transposase-mediated transgenic rescue approach [54]. The *ubiquitin* promoter (driving ubiquitous expression) and the coding sequence of *topbp1* ^*WT*^ or *topbp1* ^*cas003*^ followed by P2A peptide-mCherry fusion protein (P2A peptide allows self-cleavage of transgenesis efficacy indicator-mCherry without affecting TopBP1 protein) were constructed into the plasmid containing Tol2 arms (hereinafter referred to as *ubi*: *topbp1* ^WT^ and *ubi*: *topbp1* ^*cas003*^, Fig 3H) [55,56]. After co-injection with Tol2 transposase mRNA and *ubi*: *topbp1* ^WT^ or *ubi*: *topbp1* ^*cas003*^ constructs into one cell stage mutant^*cas003*^ embryos, *ubi*: *topbp1* ^WT^ driven ectopic expression of wild-type *topbp1* could rescue mutant^*cas003*^ phenotype at 5dpf (Fig 3I–3K), but not the *ubi*: *topbp1* ^*cas003*^ construct (Fig 3I–3J and 3L). Taken together, the MO phenocopy assays and the wild-type *topbp1* rescue assays revealed that the nonsense mutation in *topbp1* was the causative mutation in mutant^*cas003*^. Meanwhile, we changed the name of mutant^*cas003*^ into *topbp1* ^*cas003*^.

### Apoptotic HSPCs are enriched in the CHT of topbp1^*cas003*^ mutants

**Fig 4:**
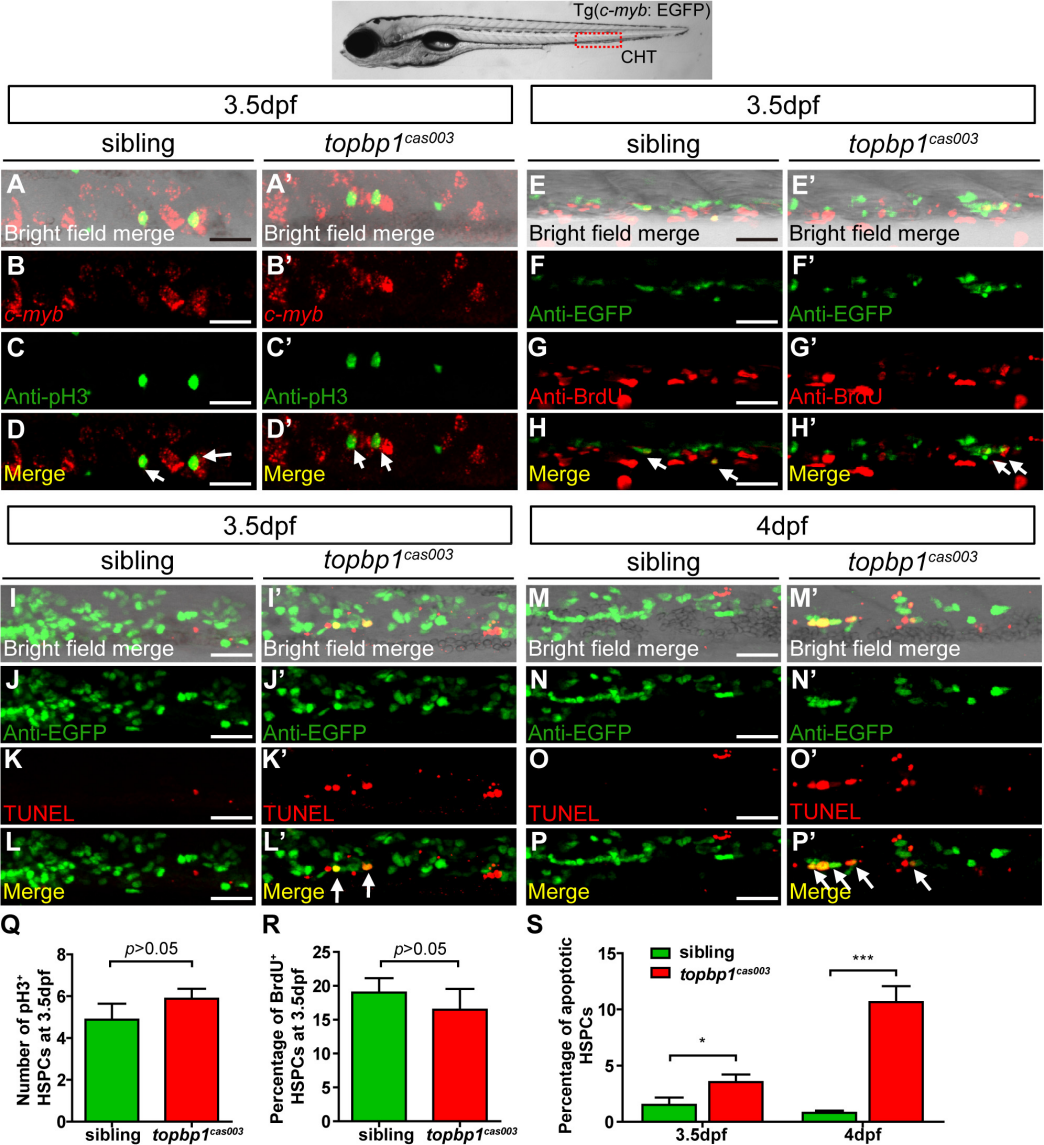
The defect of definitive hematopoiesis is due to the increased apoptosis in the HSPCs of topbp1^*cas003*^ mutants. (A-D’) The *c-myb in situ* hybridization and phospho-histone H3 (pH3) immunostaining at 3.5dpf in sibling and *topbp1* ^***cas003***^ mutant embryos, indicating no significant difference in the number of pH3^***+***^ HSPCs (pH3 and *c-myb* double positive cells, white arrows) between wild-type sibling and *topbp1* ^***cas003***^ mutant embryos. The images show the stain in the enlarged CHT region. Scale bars represent 25μm. (E-H’) Double immunostaining of *c-myb*: EGFP and BrdU at 3.5dpf in the sibling and *topbp1* ^***cas003***^ mutant embryos. The number of BrdU^***+***^ HSPCs (BrdU and EGFP double positive cells, white arrows) in *topbp1* ^***cas003***^ mutant embryos is comparable to that in wild-type siblings. Scale bars represent 25μm. (I-P’) Double immunostaining of *c-myb*: EGFP and TUNEL at 3.5dpf (I-L’) and 4dpf (M-P’) in the sibling and *topbp1* ^***cas003***^ mutant embryos. The apoptotic HSPCs (TUNEL and EGFP double positive cells, white arrows) are increased in the *topbp1* ^***cas003***^ mutants comparing to siblings, which is more profound at 4dpf. Scale bars represent 25μm. (Q) Quantification of the number of the pH3^***+***^ HSPCs in sibling and *topbp1* ^***cas003***^ mutant embryos at 3.5dpf. (R) Statistics result of the percentage of BrdU^***+***^ HSPCs at 3.5dpf. (Q) and (R) indicate no significant difference in the number of proliferating HSPCs between *topbp1* ^***cas003***^ mutant embryos and siblings at 3.5dpf. (S) Quantitative analysis of apoptotic HSPCs in the CHT region in sibling and *topbp1* ^***cas003***^ mutant embryos at 3.5dpf and 4dpf, showing the increased apoptotic HSPCs in the *topbp1* ^***cas003***^ mutant embryos. For the Quantitative analysis, at least 6 embryos were analysis for each experimental group. Error bars represent SEM. *, *p*<0.05; ***, *p*<0.001.

To explore how *topbp1* affected maintenance of HSPCs in CHT region, we first investigated the expression pattern of *topbp1* during embryonic development. WISH analysis data indicated that *topbp1* was a maternal mRNA, and was ubiquitously expressed during embryogenesis (S5A–S5J Fig). Previous reports had showed that *topbp1* knock-out or knock-down could result in either cell proliferation blockage or cell apoptosis activation [44,45]. To investigate the cause of HSPCs abrogation, we conducted cell biology assessment of HSPCs in *topbp1* ^*cas003*^ mutants in Tg(*c-myb*: EGFP) transgenic background. Double staining of *c-myb* and phospho-histone 3 (pH3) showed no significant difference in *topbp1* ^*cas003*^ mutants, compared with siblings at 3.5dpf (Fig 4A–D’, quantified in Q), suggesting that the cell cycle of HSPCs was not affected in *topbp1* ^*cas003*^ mutants. Furthermore, we performed 5-bromo-2-deoxyuridine (BrdU) incorporation assay on HSPCs, BrdU and EGFP double immunostaining results indicated that there was no significant difference in the percentage of BrdU^+^ HSPCs between siblings and *topbp1* ^*cas003*^ mutants at 3.5dpf (Fig 4E–H’, quantified in R). However, TUNEL assay showed a significant increase of apoptotic EGFP^+^ HSPCs in CHT region of *topbp1* ^*cas003*^ mutants, compared with that in wild-type siblings at 3.5dpf (Fig 4I–L’, quantified in S). At 4dpf, the percentage of apoptotic EGFP^+^ HSPCs was even more significantly increased in *topbp1* ^*cas003*^ mutants in comparison with siblings (Fig 4M–P’, quantified in S), while the number of EGFP^+^ HSPCs were dramatically decreased (Fig 2N and 2Q). Notably, we could also detect the increased apoptosis in the cranial region and the neural tube in the *topbp1* ^*cas003*^ mutants at 3.5dpf and 4dpf. Collectively, we concluded that the increased apoptosis in HSPCs was linked to the defective hematopoiesis in *topbp1* ^*cas003*^ mutants.

### Apoptosis in the HSPCs of *topbp1* ^*cas003*^ mutants is p53-dependent

**Fig 5:**
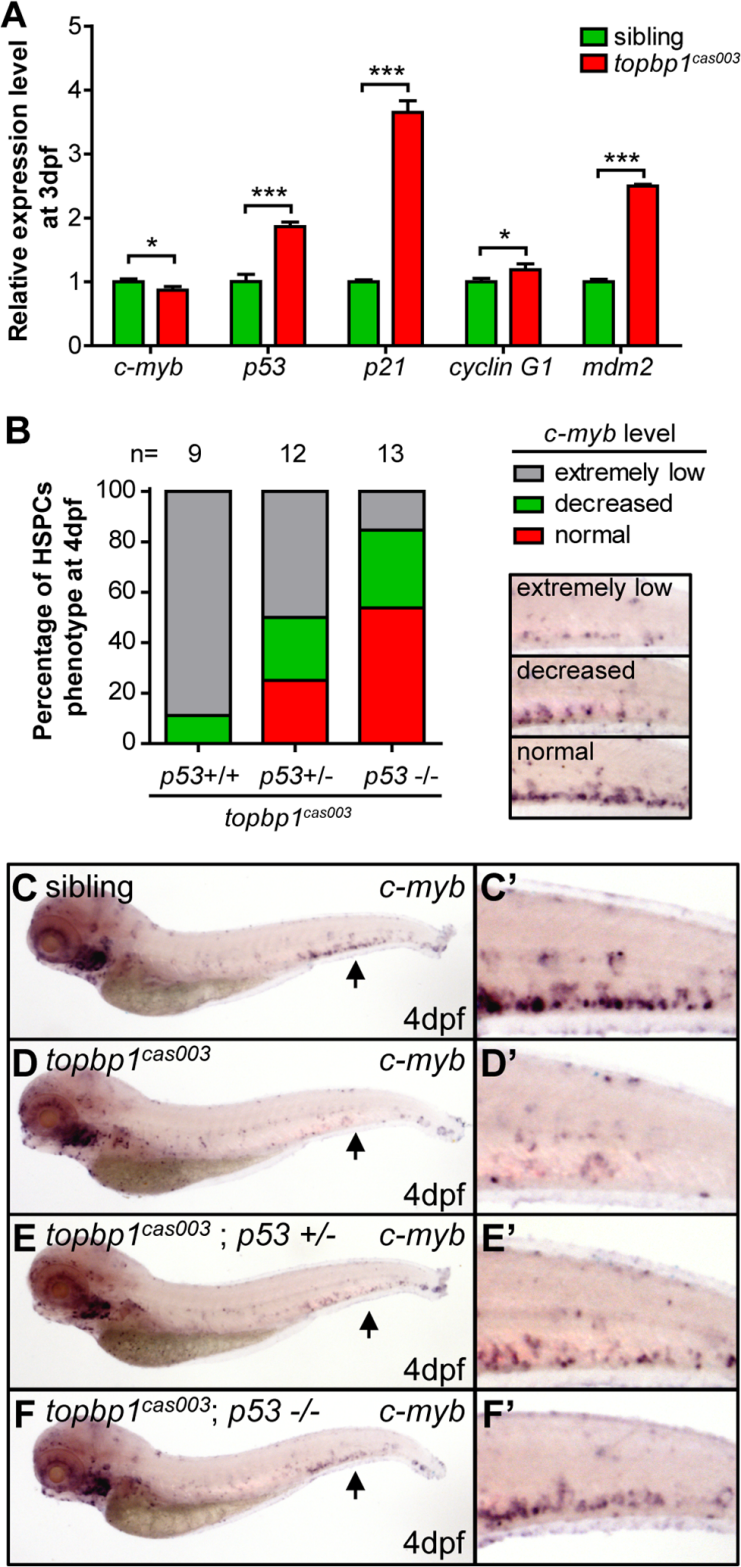
The apoptosis of HSPCs is p53 dependent in *topbp1* ^*cas003*^ mutants. (A) Quantitative PCR results of *c-myb*, *p53*, *p21*, *cyclin G1* and *mdm2* in the CHT region of wild-type sibling and *topbp1* ^***cas003***^ mutant embryos at 3dpf. All these *p53* dependent apoptosis-related genes are upregulated. Error bars represent SEM. *, *p*<0.05; ***, *p*<0.001. (B) Quantitative analysis of HSPCs phenotype, monitored by *c-myb* WISH analysis, showing inactivation of *p53* can partially rescue *c-myb* expression in *topbp1* ^***cas003***^ mutants. The 3 kinds of standards of *c-myb* WISH results in the CHT at 4dpf are shown in the right bottom. The numbers of embryos are shown above the columns. (C-F’) *c-myb* WISH analysis of siblings, *topbp1* ^***cas003***^, *topbp1* ^***cas003***^; *p53* ^***+/-***^ and *topbp1* ^***cas003***^; *p53* ^***-/-***^ embryos at 4dpf, which are quantified in B. E and E’ represent partially rescued embryos; F and F’ represent well rescued embryos. (C-D’, C-D’) 23/25 siblings and 8/9 *topbp1* ^***cas003***^ mutants show indicated phenotype. (C’-F’) Enlarged views of CHT region indicated by arrows in the left column.

To determine how TopBP1 deficiency triggered apoptosis, we firstly checked the expression of several apoptosis-related genes in the CHT regions of *topbp1* ^*cas003*^ mutants at 3dpf. The quantitative PCR results showed that the expression of *p53*, *p21*, *cyclin G1* and *mdm2* were upregulated in the CHT region of *topbp1* ^*cas003*^ mutants, indicating the p53 signaling pathway was activated (Fig 5A). Furthermore, we employed ectopic expression of Bcl2 into *topbp1* ^*cas003*^ mutants, which was known to inhibit p53 dependent apoptosis pathway [57]. WISH analysis on *c-myb* expression showed that *bcl2* mRNA could partially restore the *c-myb* expression in the CHT regions of *topbp1* ^*cas003*^ mutants (25 out of 43 embryos were partially rescued, S6B–S6D’ Fig, quantified in S6A Fig). To confirm the apoptosis in *topbp1* ^*cas003*^ HSPCs mainly depended on the p53 pathway, we crossed *topbp1* ^*cas003*^ mutant with the *tp53* ^M214K^ mutant (abbreviated as *p53* ^-/-^ below), which had been reported to abrogate p53 function in apoptosis [58]. Further investigation showed that the expression of *c-myb* in *topbp1* ^*cas003*^ mutants was partially rescued in *p53* ^+/-^ heterozygous background at 4dpf (3/12 embryos were well rescued, 3/12 embryos were partially rescued, Fig 5B–F’), and the rescue effect was more obviously in *p53* ^-/-^ background at 4dpf (7/13 embryos were well rescued, 4/13 embryos were partially rescued, Fig 5B–F’). Taken together, we concluded that the apoptosis of HSPCs in *topbp1* ^*cas003*^ mutants was p53-dependent.

### Mislocalized TopBP1^*cas003*^ causes hematopoiesis defects

**Fig 6:**
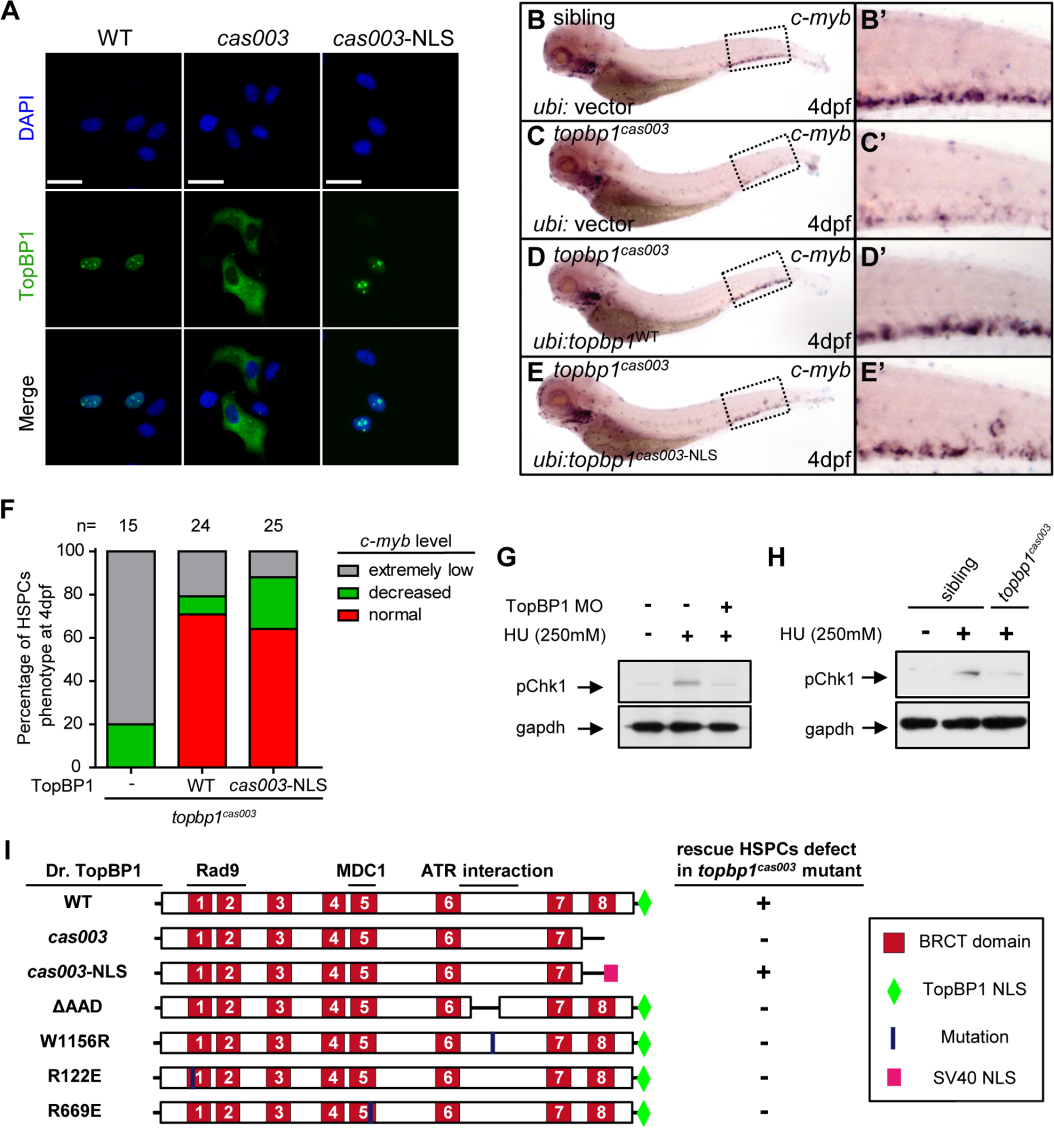
Subcellular mislocalization and defective ATR/Chk1 activation link to defects in HSPCs in the *topbp1* ^*cas003*^ mutants. (A) Confocal imaging analysis of anti-FLAG immunostaining (green) and DAPI (blue) in HeLa cells transfected with FLAG tagged TopBP1^***WT***^ (WT), TopBP1^***cas003***^ (*cas003*) and TopBP1^***cas003-NLS***^ (*cas003*-NLS). TopBP1^***WT***^ is mainly localized in the nucleus (left column), cytosol mislocalization of TopBP1^***cas003***^ (middle column) can be corrected by the additional SV40 NLS on its C-terminus (TopBP1^***cas003-NLS***^, right column). Scale bars represent 25 μm. (B-F) WISH analysis of hematopoiesis in siblings and *topbp1* ^***cas003***^ mutant embryos with Tol2-transposase mediated *topbp1* ^***WT***^ and *topbp1* ^***cas003-NLS***^ transgenesis at 4dpf, which are quantified in F (the numbers of embryos are shown above). Defective *c-myb* expression in *topbp1* ^***cas003***^ mutants can be rescued by *topbp1* ^***cas003–NLS***^ as well as *topbp1* ^***WT***^. (B-C, B’-C’) 28/32 siblings and 12/15 *topbp1* ^***cas003***^ mutants show indicated WISH results. (D-E, D’-E’) WISH results of well rescued mutants. (B’-E’) Enlarged views of CHT region in the left column. (G) Western blot with pChk1 antibody in control morphants and hydroxyurea (HU) treated control or *topbp1* morphants. The morphants were treated with 250mM HU or mock from 60hpf to 76hpf. *topbp1* knockdown could abrogate the Chk1 phosphorylation in the tail region upon HU treatment. (H) Immunoblotting analysis showing reduced phospho-Chk1 level in tail region of *topbp1* ^***cas003***^ mutants upon HU treatment, comparing to that in wild-type siblings. The embryos were treated with 250mM HU or mock from 60hpf to 76hpf. (I) Schematic diagram of variant forms of TopBP1, including wild-type (WT), *cas003*, *cas003*-NLS, ΔAAD, W1156R, R122E and R669E mutation. The regions associated with ATR activation and Rad9 or MDC1 interactions are indicated. After Tol2-mediated transient transgenesis of variant forms of TopBP1 into *topbp1* ^***cas003***^ mutant embryos, quantitative analysis of the *c-myb* expression in the CHT region at 4dpf was performed for the evaluation of rescue capability (n>20). “+” (rescue); “-” (not rescue effect). Except WT and *cas003*-NLS, all TopBP1 mutation forms are unable to rescue hematopoietic defects in *topbp1* ^***cas003***^ mutants.

To further understand the molecular mechanism of HSPC apoptosis which was induced by this particular defective TopBP1 without its 8^th^ BRCT domain and the putative NLS domain, we analyzed the subcellular localization of TopBP1^*cas003*^. Confocal imaging showed that flag-tagged TopBP1^WT^ was predominantly localized in the nucleus of transfected HeLa cells (Fig 6A, left column). However, TopBP1^*cas003*^ was mistakenly localized in cytoplasm (Fig 6A, middle column), which was consistent with our previous sequence analysis on the lack of putative NLS in TopBP1^*cas003*^ (Fig 3C) and immunoblotting analysis on TopBP1^*WT*^/TopBP1^*cas003*^ protein in cytoplasmic and nucleus fractions of pooled embryos from heterozygote incrossing (S5L Fig). Moreover, addition of SV40 NLS at C terminus of TopBP1^*cas003*^ was sufficient to correct TopBP1^*cas003*^ subcellular localization defect (Fig 6A, right column). To test whether the hematopoietic deficiency in *topbp1* ^*cas003*^ mutants could also be rescued by the nuclear localized TopBP1^*cas003*^, we carried out transient transgenesis of *topbp1* ^*cas003*-NLS^ or *topbp1* ^WT^ (as the positive control) in the *topbp1* ^*cas003*^ mutants. WISH results of *c-myb* at 4dpf indicated that ectopic expression of *topbp1* ^*cas003*-NLS^ could rescue *c-myb* expression in *topbp1* ^*cas003*^ mutants, as efficient as transgenesis with *topbp1* ^WT^ (Fig 6B–E’, quantified in F). Collectively, we concluded that the loss of NLS in TopBP1^*cas003*^ and the failure of nuclear localization directly caused HSPCs deficiency in *topbp1* ^*cas003*^ mutants.

### Chk1 activation is reduced in *topbp1* ^*cas003*^ mutants

Previous studies have demonstrated that TopBP1 plays conserved roles as a scaffold protein that is important for DNA replication and DNA damage response (DDR) [27,29,37]. Since the proliferation of HSPCs was not disrupted in *topbp1* ^*cas003*^ mutants (Fig 4A–H’), it seemed that the function of TopBP1 in DDR instead of DNA replication was responsible for the HSPCs defect in the mutants. Firstly, we checked the activation of TopBP1-ATR-Chk1 pathway in *topbp1* ^*cas003*^ mutants and siblings under the hydroxyurea (HU) treatment, which was extensively applied to mimic DNA replication stress and could activate ATR-Chk1 axis in mammalian cells and zebrafish embryos [30,59,60]. The phospho-Chk1 (pChk1) level in CHT region was significantly increased after 250mM HU treatment from 60hpf to 76hpf (Fig 6G, lane1 and 2). However, the activation of pChk1 was abrogated in *topbp1* morphants (Fig 6G, lane 3). Consistently, we also observed dramatic ablation of pChk1 elevation in the CHT of *topbp1* ^*cas003*^ mutants compared with wild-type siblings (Fig 6H).

Furthermore, we analyzed protein-protein interaction sites in TopBP1 on the basis of previous biochemical and structural investigations [31,41–43,61,62]. The R122, R669 and W1156 sites in TopBP1 are involved in Rad9 or MDC1 interaction and ATR activation, respectively. All these sites are highly conserved among zebrafish, human and mouse (S7 Fig), and they are critical for TopBP1-ATR pathway [31,41,61,63]. Transient transgenesis of TopBP1^ΔAAD^, TopBP1^W1156R^, TopBP1^R122E^, TopBP1^R669E^ and TopBP1^WT^ (as positive control) in *topbp1* ^*cas003*^ mutants was analyzed for hematopoiesis monitored by *c-myb* WISH. None of these mutated TopBP1 could rescue the hematopoietic failure in *topbp1* ^*cas003*^ mutants, compared with TopBP1^WT^ (Fig 6I), indicating that ATR activation function of TopBP1 was essential for HSPCs survival in *topbp1* ^*cas003*^ mutants. Taken together, these data implied that the blockage of TopBP1-ATR-Chk1 pathway was correlated to the defective HSPCs in *topbp1* ^*cas003*^ mutants.

### DNA damage response is impaired in *topbp1* ^*cas003*^ mutants

**Fig 7:**
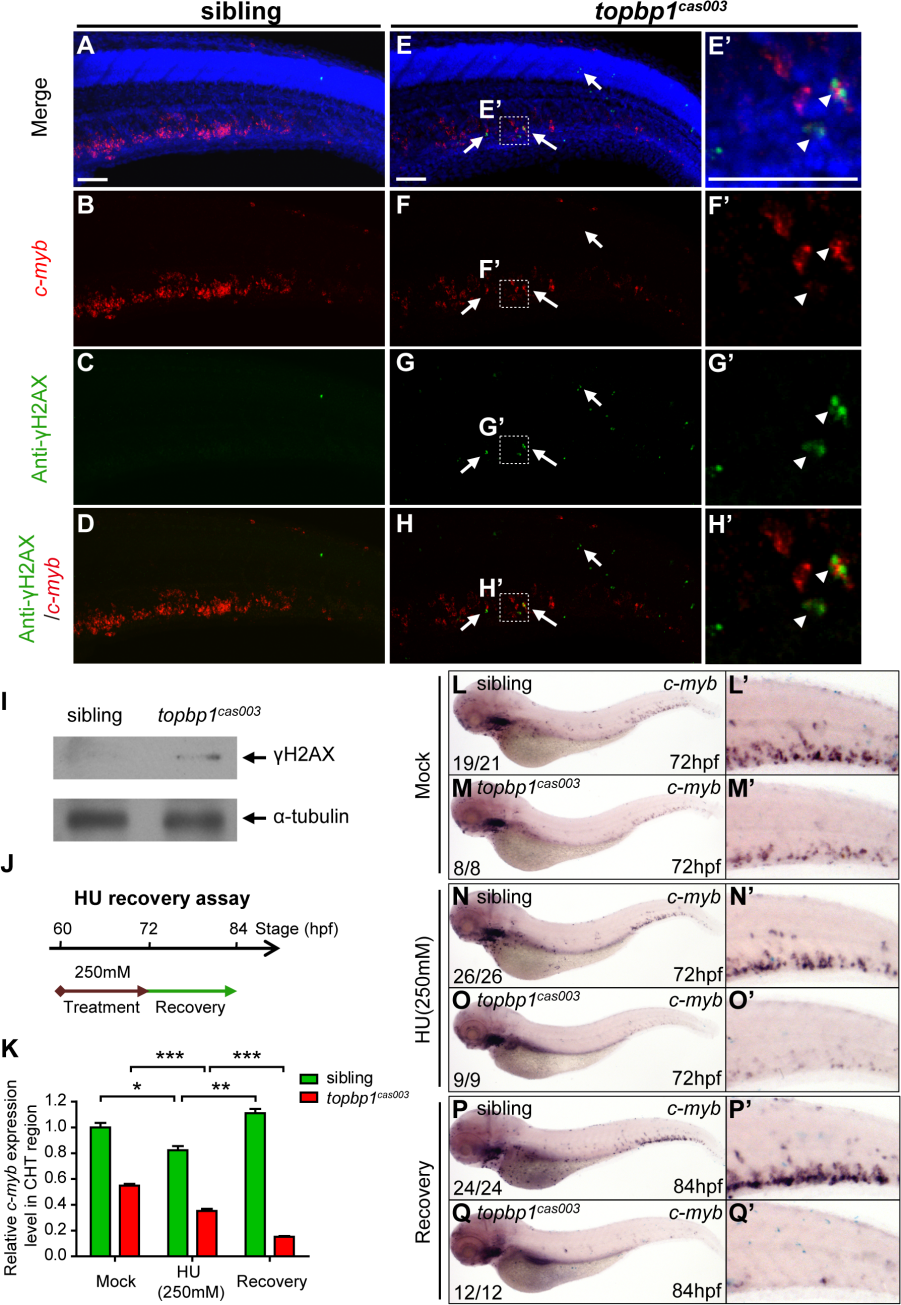
*topbp1* ^*cas003*^ mutants manifest deficient DNA damage repair capability. (A-H’) Immuno-staining of γH2AX (green), *c-myb* fluorescent *in situ* hybridization (red) and DAPI stain (blue) in sibling and *topbp1* ^***cas003***^ mutant embryos at 3.5dpf. (E’-H’) Magnified views of the regions in the dashed boxes showing γH2AX^***+***^; *c-myb* ^***+***^; DAPI^***+***^ cell in the CHT region. Arrows indicate the γH2AX^***+***^; DAPI^***+***^ cells. The γH2AX^***+***^; DAPI^***+***^ cells are increased in *topbp1* ^***cas003***^ mutants comparing to siblings. Moreover, γH2AX^***+***^; *c-myb* ^***+***^; DAPI^***+***^ cells can only be detected in *topbp1* ^***cas003***^ mutants (H’). Scale bars represent 50μm. (I) Immunoblotting showing increased γH2AX level in the CHT of *topbp1* ^***cas003***^ mutant embryos at 3dpf. (J) Procedure of the hydroxyurea (HU) recovery assay. Brown arrow line represents 250mM HU or mock treatment from 60hpf to 72hpf; green arrow line indicates removal of HU or mock treatment from 72hpf to 84hpf for recovery. (K-Q) The *c-myb* expression is further decreased in *topbp1* ^***cas003***^ mutant embryos under HU treatment. Wild-type siblings or *topbp1* ^***cas003***^ mutant embryos, after 12-hours mock treatment or after 12-hours 250mM HU treatment and sequential 12-hours recovery, were analyzed by either quantitative PCR (K) or *c-myb* WISH analysis (L-Q). *c-my*b level can be recovered in wild-type siblings, but not in *topbp1* ^***cas003***^ mutant embryos. The penetrance of the indicated phenotype is shown in the bottom left of each panel. Error bars represent SEM. * *p*<0.05; ** *p*<0.01; *** *p*<0.001. (L’-Q’) Enlarged views of the CHT region of embryos in the left columns.

Since TopBP1-ATR-Chk1 axis was disrupted in *topbp1* ^*cas003*^ mutants, the unresolved DNA replication stress would result in collapse of replication forks, which could introduce DNA double-stranded breaks ultimately [18]. To check whether the apoptosis of HSPCs was due to the accumulation of DNA damage in CHT region, we carried out fluorescent c*-myb* WISH analysis and immunostaining with phosphorylated histone H2AX (γH2AX) antibody, which was a typical DNA damage marker [64], from 39hpf to 3.5dpf. Interestingly, we couldn’t detect any γH2AX^+^ cells in AGM region at 39hpf in both *topbp1* ^*cas003*^ mutants and siblings, but γH2AX^+^ HSPCs emerged in CHT region in *topbp1* ^*cas003*^ mutants at the same stage (S8A–S8B Fig). Moreover, γH2AX^+^ HSPCs were accumulated in CHT region of *topbp1* ^*cas003*^ mutants afterward (S8C Fig), and they were obviously increased at 3.5dpf, (Fig 7A–H’, S8C Fig) indicating the DNA damage was indeed accumulated in HSPCs in *topbp1* ^*cas003*^ mutant. In addition, we could also observed several γH2AX^+^ cells in neuronal tissue (Fig 7G), which was consistent with previous investigation [45]. Furthermore, the immunoblotting of γH2AX within CHT regions of *topbp1* ^*cas003*^ mutants at 3dpf also showed an increase of DNA damage (Fig 7I). Collectively, we found that DNA damage was accumulated in HSPCs in *topbp1* ^*cas003*^ mutants.

To further examine whether the hematopoietic failure was due to the defective DDR upon DNA replication stress in *topbp1* ^*cas003*^ mutants, we challenged the embryos with HU. Indeed, high concentration treatment from 52hpf to 76hpf directly caused embryonic lethality in *topbp1* ^*cas003*^ mutants (over 65%), however the effect on wild-type siblings was much milder (<15%) (S9A and S9B Fig) [60]. Furthermore, we carried out a recovery assay in the HU-treated zebrafish embryos (Fig 7J). Interestingly, despite of a suppression by HU treatment, the *c-myb* expression was recovered in wild-type sibling embryos after challenge removal (Fig 7K, 7L–L’, 7N–N’ and 7P–P’). In contrast, the *c-myb* expression level was not recovered, but decreased further in the HU-treated *topbp1* ^*cas003*^ mutant embryos (Fig 7K, 7M–M’, 7O–O’ and 7Q–Q’). Taken together, all these observations suggested that the HSPCs in CHT of *topbp1* ^*cas003*^ mutants were defective in replicative DNA damage response and they eventually underwent apoptosis through a p53-dependent signaling pathway.

## Discussion

In this study, we reported a novel zebrafish mutant *topbp1* ^*cas003*^, which manifested severe defect in definitive hematopoiesis. The reduction of HSPCs started from 3dpf, which was mainly due to the increased p53*-*dependent apoptosis, rather than proliferation deficiency. Genetic assessment revealed that a nonsense mutation in *topbp1* gene was causative for the hematopoiesis failure. Further investigation revealed that the mutated TopBP1^*cas003*^ protein was decreased and mislocalized from nucleus to cytoplasm which compromised the DNA damage response. As a result, it led to accumulated DNA damage that triggered sequential apoptosis of HSPCs in *topbp1* ^*cas003*^ mutants.

**Fig 8:**
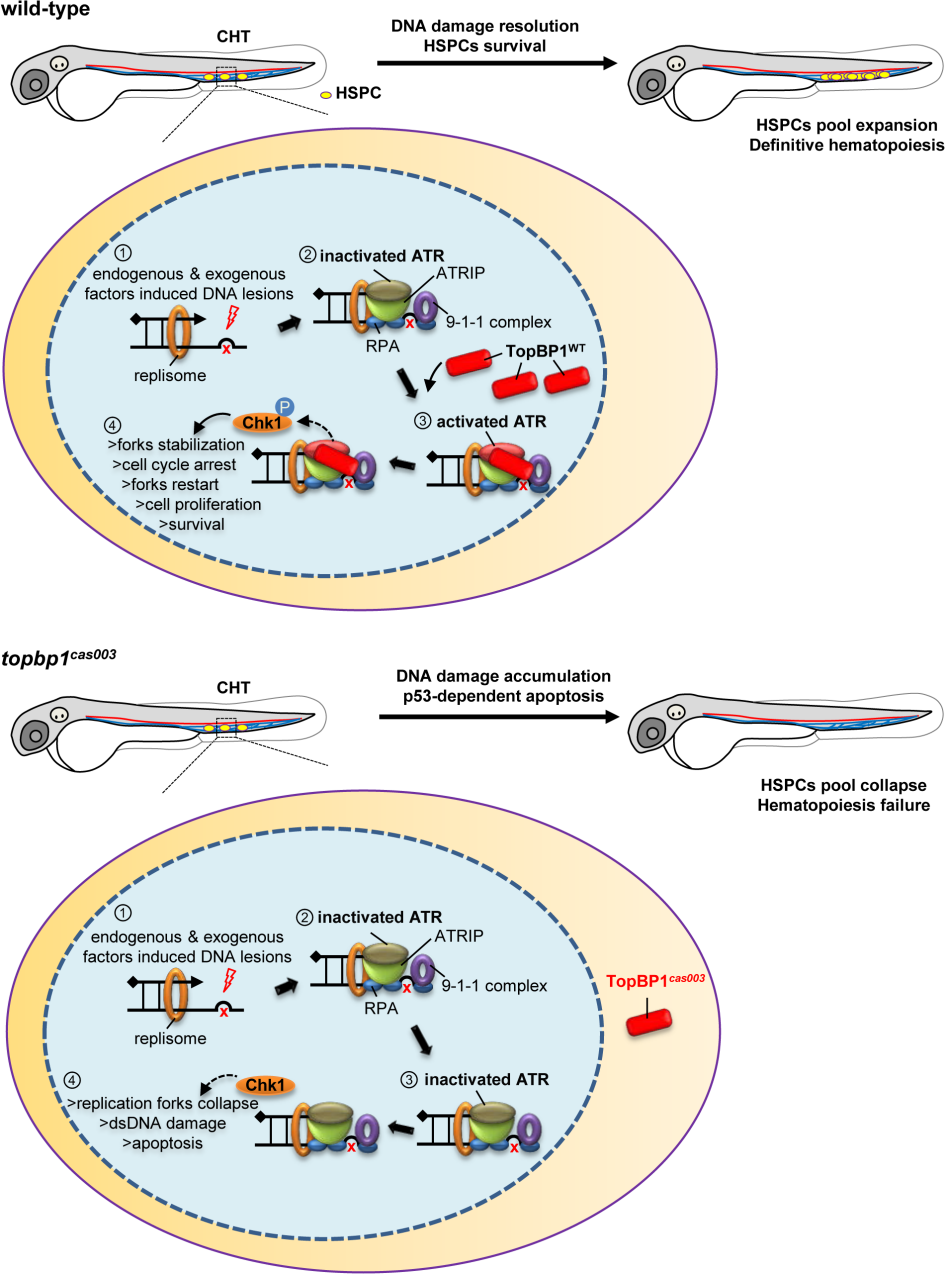
TopBP1 governs HSPCs survival during pool expansion in CHT. In zebrafish embryogenesis, the nascent HSPCs undergo extensive pool expansion in CHT after 2dpf, while the replication stress causes DNA damage. In normal HSPCs, the stalled replication forks will generate the typical dsDNA-ssDNA structure. After the loading of replication protein A (RPA), ATR/ATRIP, and 9-1-1 complex, sequential participation of TopBP1 can largely activate ATR kinase activity, and the latter phosphorylate downstream targets such as Chk1. The pChk1 can stabilize replication forks and arrest the cell cycle in order to give more time for DNA damage repair for the replication fork restart. As a result, the HSPCs can survive and go through the pool expansion continuously. In *topbp1* ^***cas003***^ HSPCs, TopBP1^***cas003***^ decreases and mislocalizes in cytosol. ATR and Chk1 are prevented from activation in the circumstance of replication stress. Moreover, the stalled replication forks may collapse and trigger p53-dependent apoptosis, which finally results in hematopoiesis failure.

In zebrafish definitive hematopoiesis, HSPCs undergo extensive proliferation in the CHT region around 3dpf, during which the replication stress, characterized by the stalled replication forks, can be induced by various endogenous and exogenous factors [18]. The stalled replication forks will generate typical dsDNA-ssDNA structure, followed with proper loading of RPA, ATR-ATRIP and 9-1-1 complex [37]. Sequential recruitment of TopBP1 can largely activate ATR kinase activity, and the latter will phosphorylate downstream molecules including Chk1. Activated Chk1 stabilizes the replication forks and arrests cell cycle in order to leave enough time for DNA damage repair machinery to work and to restart the replication fork, so that HSPCs can survive the stress and finish their pool expansion (Fig 8).

The quantitative analysis and WISH results demonstrated that nonsense mutation in *topbp1* might lead to nonsense mediated mRNA decay. The expression level of *topbp1* was decreased over 80% in the whole embryo and about 50% in CHT of *topbp1* ^*cas003*^ mutants (S5M–S5Q Fig). Although around 50% TopBP1^*cas003*^ protein remains in CHT, it was mistakenly localized in cytosol, while TopBP1^*WT*^ was mainly in nucleus to play its role in DDR (S5L Fig). Our results suggest that TopBP1^*cas003*^ is decreased and fails in its nucleus entry due to the loss of its C-terminal NLS, abrogating the later ATR/Chk1 activation. In *topbp1* ^*cas003*^ HSPCs, the unresolved stalled replication forks would collapse and generate multiple DNA fragile sites, which can induce dsDNA break [18]. As a result, p53-dependent apoptosis is elevated in *topbp1* ^*cas003*^ HSPCs, impairing the HSPCs pool severely (Fig 8).

Recently an improved clustered regularly interspaced short palindromic repeats (CRISPR)/ CRISPR-associated proteins (Cas9) system with custom guide RNAs (gRNAs) and a zebrafish codon-optimized Cas9 protein showed high mutagenesis rate in zebrafish, which could even generate biallelic mutations in the F0 generation [65,66]. In order to confirm that the deficiency of TopBP1 could disrupt the development of HSPCs, we adapted this optimized CRISPR/Cas9 system to obtain other *topbp1* zebrafish mutants (S10A–S10B Fig). Some of the *topbp1* Cas9 injected wild-type embryos displayed dramatically decreased *c-myb* expression as same as *topbp1* ^*cas003*^ mutant at 4dpf (S10C–S10D’ Fig). And this phenotype could be reached in higher efficiency when the injected embryos were generated from the outcross between *topbp1* ^*cas003*^ heterozygote and wild-type fish (S10E–S10F’ Fig). Conclusively, these data provided additional evidence that definitive HSPCs were defective in the TopBP1 loss-of-function embryos.

It is an intriguing finding that *topbp1* plays an essential role in proliferative tissues, especially in the definitive hematopoiesis without affecting the morphogenesis at the early stage, whereas its transcripts were ubiquitously distributed in the embryogenesis (S5 Fig), and TopBP1 knockout mice were reported to be lethal at the peri-implantation stage [44]. The WISH analysis showed maternal expression of *topbp1* (S5A Fig), suggesting that homozygote *topbp1* mutant embryos can inherit wild-type *topbp1* mRNA from the female parents to support its early development until zygotic *topbp1* expresses latter in the development. Nevertheless, we attempted to figure out whether *topbp1* was expressed and functional in the HSPCs. Quantitative PCR analysis on the CD41^+^ cell population in the tail region of Tg(*CD41*: EGFP) embryos, which was reported to be an enriched population of HSPCs at 5dpf [67,68], showed that the level of *topbp1* mRNA was 3-fold enriched in CD41^+^ cells, compared to cells in the whole tails, demonstrating its expression in HSPCs (S5K Fig) [5]. Furthermore, due to the lack of definitive hematopoiesis-specific promoter, we used hemangiogenic promoter *lmo2*, which was also expressed in definitive HSPCs, to drive the ectopic expression of wild-type *topbp1* into *topbp1* ^*cas003*^ mutants [52,69], we could indeed observe the expression of mCherry driven by *lmo2* promoter in CHT region at 5dpf, and this construct could partially rescue the HSPCs deficits at 5dpf (S11 Fig). In addition, the vascular plexus in CHT region was normal in *topbp1* ^*cas003*^ mutants or morphants from 2dpf to 5dpf (S1E–S1L Fig and S4O–S4R Fig), and low dose microinjection of *topbp1* morpholino was sufficient to induce definitive hematopoiesis deficits in CHT without affecting the primitive hematopoiesis and vascular system in wild-type embryos (S3C–S3D Fig, S4 Fig). Taken all these data together, we concluded that TopBP1 played an essential and HSPC-intrinsic mechanism during definitive hematopoiesis.

It is intriguing that whether the truncated TopBP1 can potentially function as a dominant negative protein. Ectopic expression of *cas003* mutant form of TopBP1 (TopBP1^*cas003*^) driven by *ubiquitin* promoter was performed in wild-type fish, and it did not cause defective definitive hematopoiesis (S12 Fig). The possible reason for this phenomenon was that the mutated TopBP1 could not enter nucleus to compete with wild-type TopBP1. Meanwhile, the hematopoietic phenotype of *topbp1* ^*cas003*^ heterozygotes was checked, and no HSPCs defect was observed. Taking these results together, we concluded that TopBP1^*cas003*^ could not function as a dominant negative form.

In definitive hematopoiesis, nascent HSPCs seldom proliferate in AGM region, but they become active in cell cycle and undergo extensive proliferation in CHT region supported by niche cells, meanwhile, they have to overcome DNA replicative stress [13,15,18]. BrdU incorporation assays within Tg(*c-myb*: EGFP) embryos confirmed that HSPCs underwent high proliferation at a constant rate from 2dpf to 5dpf, although the expansion of neural tube cells was gradually attenuated (S13 Fig). As a result, the defect in HSPCs was more profound after 3dpf in the *topbp1* ^*cas003*^ mutant. Consistently, we indeed found obvious accumulation of γH2AX positive cells (2.5dpf) and increased apoptotic cells (3.5dpf) in cranial and neuron tube tissues of *topbp1* ^*cas003*^ mutant, which was in agreement with previous observations in neuron-specific TopBP1 knock-out mice [45]. Besides, some of homozygote *topbp1* mutant embryos developed smaller head and eyes after 6dpf, and all of them eventually died around 10–20 dpf.

Previous works within zebrafish mutants revealed several genes and pathways which were critical for the HSPCs development in CHT region, including genes involved in mitotic spindle assembly, maintenance of centrosome integrity and mitotic progression; pre-mRNA processing; sumoylation of genes participating in DNA replication or cell cycle regulation [5,70,71]. All these genes were indispensable for cell to complete proliferation or division. Because the HSPCs were highly proliferative in CHT, these data depict a picture that the HSPCs in fetal stage are extremely sensitive to the disruption of genes participating in various processing to complete cell division successfully and faithfully. As the DDR pathway is essential for genomic fidelity and stability during DNA replication, our work revealed that DDR pathway is also critical for HSPCs development in fetal stage.

It has been reported that Fanconi anemia pathway is critical for the repair of DNA cross-link damage [26]. Biallelic mutations in any of 15 FANC genes will result in Fanconi anemia (FA), which can most frequently develop into inherited bone marrow failure (BMF) syndrome [72]. The work of Raphael Ceccaldi et al. revealed that the FA patients showed profound HSPCs defect before the onset of BMF [73]. The *p53-p21* axis, triggered by replicative stress, was highly elevated in FA HSPCs, and the *p53* silence can rescue hematopoietic deficits [73]. They also pointed out that *p53* activation, caused by unresolved cellular abnormality, may be the signaling mechanism for inherited BMF, and the *p53* activation was commonly found in other types of inherited BMF syndromes, such as Diamond Blackfan anemia (DBA) and dyskeratosis congenital (DC) [73]. HSPCs in *topbp1* ^*cas003*^ mutants manifested similar features as that in FA (Fig 5), whether *topbp1* could be a putative pathogenic gene in human BMF syndrome needs further investigation.

Zebrafish *fancd2* morphant exhibited developmental abnormalities and p53-dependent apoptosis, however its hematopoietic phenotype had not been extensively investigated [57]. The *emi1* homozygous mutants showed disrupted genomic integrity and hematopoiesis failure [74]. Studies on *topbp1* ^*cas003*^ mutants revealed that DNA damage and apoptosis signaling was accumulated in the HSPCs *of topbp1* ^*cas003*^ homozygous embryos, which linked to the hyper-activated *p53*-*p21* axis (Fig 5) and failed ATR/Chk1 activation (Fig 6). Furthermore, TopBP1-involved *c-myb* regulated DDR pathway was proposed by recent studies on castration-resistant prostate cancer [75]. HU treatment of the developing zebrafish further emphasized the importance of DNA damage response and repair pathway for HSPCs survival during high proliferation stage.

Collectively, we demonstrated a novel and essential role of TopBP1 in HSPCs during their rapid proliferation in fetal hematopoiesis. Due to the dramatic definitive hematopoiesis phenotype in embryogenesis, *topbp1* ^*cas003*^ mutants provide a unique model for the mechanism study and small molecular chemical screen on BMF-like hematopoiesis failure, which is caused by defective replicative DNA damage response.

## Materials and Methods

### Ethics statement

The zebrafish facility and study were approved by the Institutional Review Board of the Institute of Health Sciences, Shanghai Institutes of Biological Sciences, Chinese Academy of Sciences (Shanghai, China), and zebrafish were maintained according to the guidelines of the Institutional Animal Care and Use Committee.

### Zebrafish maintenance and manipulation

Wild-type (WT) zebrafish strains Tubingen (TU) and WIK, the transgenic zebrafish line Tg(*c-myb*: EGFP) [52], Tg(*fli1*: EGFP) [50], Tg(*CD41*: EGFP) [76], the mutant zebrafish line *tp53* ^M214K/M214K^ [58] were maintained as previously described [77]. For the forward genetics screen, WT TU zebrafish line was treated with ethylnitrosourea (ENU, Sigma) to generate mutants, the screen approach was performed as previously described [78,79]. The desired mutants within F3 generation were identified by the whole-mount *in situ* hybridization (WISH) using *c-myb* probe at 5dpf. For the chemical treatment, the hydroxyurea (HU, Sigma) was dissolved with distilled water into 1M and stored at -20℃. The embryos were treated with 250mM HU as the indicated procedures in the egg water at 28.5℃ [59,60]. To prevent the formation of melanin pigment, the embryos were incubated in egg water containing 0.045% 1-phenyl-2-thiourea (PTU, Sigma) after gastrulation stage. The embryos were collected at the desired stages [80].

### Positional cloning and genotyping of mutant^*cas003*^

Positional cloning was carried out with WIK line as previously described [81]. Firstly, the mutation was mapped to chromosome 24 by bulk segregation analysis (BSA) with simple sequence length polymorphism (SSLP) markers. Through high resolution mapping analysis on 1041 mutants, the mutation was finally flanked by two SSLP markers, L0310_5 and R0310_4. The candidate genes in this range were sequenced with wild type sibling and mutant cDNA, and the putative mutation was confirmed by genomic DNA sequencing. The primers used in the positional cloning were provided in supplemental S1 Table. Most experiments in this study were carried out with the embryos generated by the incross of mutant^*cas003*^ heterozygote pairs (TU/WIK background) used in the positional cloning if possible. The mutants can be identified by flanked SSLP markers, such as Z9852 and R0306_4. Alternatively, the mutants can be distinguished by restriction fragment length polymorphism (RFLP) using EcoP15I (NEB), the RFLP primers were provided in supplemental data (S1 Table).

### Plasmid construction

To construct Tol2 transgenesis vectors, the *ubiquitin* promoter [55] or *lmo2* promoter [69] followed by P2A [56] and in-frame mCherry was cloned into modified Tol2 backbone [82]. The vectors were referred as p*Ubi*-Tol2 or p*Lmo2*-Tol2 below. The genes of interest can be inserted between the promoter and P2A. Zebrafish *topbp1* ^WT^ or *topbp1* ^*cas003*^ were amplified and inserted into p*Ubi*-Tol2 or p*Lmo2*-Tol2 vectors. To generate the mutated forms of *topbp1*, the mutagenesis was carried out following QuikChange mutagenesis kit instruction using p*Ubi-topbp1* ^WT^-Tol2 plasmid as the template. The region of TopBP1 (984–1206) are the putative ATR activation domain (AAD) between BRCT6 and BRCT7. In TopBP1^ΔAAD^, the coding sequence of TopBP1 (1083–1159) containing conserved RQLQ and WDDP sequences are deleted [31]. The fragment of *topbp1* (-9–692) was amplified and inserted into the pCS2+ vector for *in situ* probe preparation. To construct *topbp1* MO effect evaluation plasmid, a DNA fragment containing *topbp1* ATG MO targeting site was inserted into the upstream of EGFP coding region in pCS2+. Zebrafish *topbp1* ^WT^and *topbp1* ^*cas003*^ were cloned into pCMV4-FLAG-4 vector (Sigma). The SV40 NLS (nuclear localization signal) sequence (5’-CCAAAAAAGAAGAGAAAGGTA-3’) [83] was firstly cloned into pCMV4-FLAG-4 vector in the 3’ end of FLAG tag, and then the *topbp1* ^*cas003*^ sequence was inserted into the pCMV4-FLAG-NLS plasmid. All of the primers used were listed in S1 Table.

### Microinjection and Cas9 mutagenesis

The mRNA was synthesized *in vitro* by SP6 mMessage mMachine Transcription Kit (Ambion). The *topbp1* gRNA was synthesized as described [66]. The information of the *topbp1* gRNA target site was shown in S1 Table. The zebrafish optimized Cas9 mRNA was synthesized *in vitro* from the pCS2-nCas9n plasmid (addgene, #47929) as described [65]. *bcl2-*egfp mRNA (~100pg) was injected into 1-cell stage embryos [54]. For the ectopic-expression, Tol2 transposon-mediated transient transgenesis was applied and performed as previously described [84]. A series of *topbp1* transgene constructs within Tol2 vectors (~40 ng/μl) were mixed with transposase mRNA (~60 ng/μl) and 0.2 M KCl, and then injected into 1-cell stage embryos, respectively [85]. The volume of the mixture injected was about 0.5nL. The *topbp1* ATG morpholino oligo (MO) (5’-CCTTGCTGGCTTTCGACATGGTGAC-3’) and control morpholino (5’- CCTCTTACCTCAGTTACAATTTATA-3’) were synthesized by Gene Tool company and was injected into 1-cell stage embryos. For Cas9 assay, *topbp1* gRNA (50pg) and Cas9 mRNA (150pg) were co-injected into one-cell stage embryos. The T7EI assay was performed as described [65].

### WISH and immuno-fluorescence double staining

*c-myb*, *runx1*, *ae1-globin*, *mpx*, *lyz*, *rag1* and *topbp1* probes were transcribed *in vitro* by T3 or T7 polymerase (Ambion) with Digoxigenin RNA Labeling Mix (Roche). One color WISH was performed as described previously [54]. Images were photographed by the Nikon SMZ1500 microscope with Nikon DXM 1200F CCD or Olympus SZX16 microscope with Olympus DP80 CCD. *c-myb* RNA and immuno-fluorescence double staining was carried out as described previously [70]. For the immunostaining, rabbit anti-pH3 antibody (1:500, Santa Cruz) and rabbit anti-γH2AX antibody (1:500, gift from Dr. James Amatruda, University of Texas Southwestern) were used. The embryos were stained with goat-Alexa Fluore488-conjugated anti-rabbit secondary antibody (1:500, Invitrogen). DAPI (1:500, Beyotime) staining was carried out along with the secondary antibody incubation if necessary.

### BrdU incorporation and TUNEL immunostaining

The 3.5dpf topbp1^*cas003*^ mutant/Tg(*c-myb*:EGFP) or sibling embryos were soaked in egg water containing 10mM BrdU (Sigma)/15% DMSO for 30 minutes at 28.5℃ or injected with 1nL 30mM BrdU into the yolk sac. Then they were transferred into fresh egg water and incubated for 2 hours. After fixation in 4% paraformaldehyde (PFA, Sigma), the embryos were dehydrated with methanol and stored at -20℃ overnight. For BrdU immunostaining, the rehydrated embryos were digested with Proteinase K(12 μg/ml, Roche) at 30℃ for 28 minutes and treated with acetone at -20℃ for 30 minutes. After re-fixation with 4% PFA, the embryos were blocked with the block solution (PBS + 0.3% Triton-X -100 +1% DMSO+ 10 mg/ml BSA+10% normal goat serum) for 2 hours at RT. The embryos were then incubated with anti-GFP Rabbit Serum (1:500, Invitrogen) followed by goat-Alexa Fluore488-conjugated anti-rabbit secondary antibody (1:500, Invitrogen) incubation. 2N HCl was used to treat the embryos for 1 hour at room temperature (RT). After that, the embryos were stained with mouse anti-BrdU primary antibody (1:50, Roche) and goat-Alexa Fluore546-conjugated anti-mouse secondary antibody (1:500, Invitrogen). TUNEL assay was performed with the In Situ Cell Death Detection Kit TMR red (Roche). Similar to the BrdU immunostaining, 3.5dpf and 4dpf topbp1^*cas003*^ mutant/Tg(*c-myb*:EGFP) or sibling embryos were fixed with 4% PFA. After methanol dehydration, rehydration, Proteinase K digestion and acetone treatment, the embryos were permeated with permeabilisation solution (0.5% Triton X–100, 0.1% sodium citrate in PBS) at RT for 4 hours. Then the embryos were stained with the TUNEL Kit (100ul, enzyme: labeling solution = 1:9) at 37℃ for 2 hours. Finally, the EGFP immunostaining was carried out as described above.

### Cell sorting, RNA extraction and quantitative PCR

The CD41^+^ cells were sorted from the tails of Tg(CD41: EGFP) embryos including the CHT region at 5dpf as previously described [86,87]. The total RNA was extracted from TRIzol (invitrogen) dissolved zebrafish whole embryos or the tails including CHT region or the sorted cells, and then transcribed into cDNA by PrimerScript RT Master Mix (TaKaRa). The quantitative PCR was carried out with SYBR Green Real-time PCR Master Mix (TOYOBO) with ABI 7900HT real-time PCR machine, and analyzed with Graphpad 5.1 software. The primers used were listed in S1 Table.

### Cell culture, plasmid transfection and immunostaining

HeLa and HEK293T cells were maintained in DMEM with 10% Fetal Bovine Serum (FBS) and penicillin-streptomycin antibiotics (1:100). Plasmid transfection was carried out with Lipofectamine 2000 (Invitrogen) according to manufacturer’s instruction. The immunostaining was carried out in HeLa cells as previously described [70]. FLAG-*topbp1* ^WT^, FLAG-*topbp1* ^*cas003*^ and FLAG-*topbp1* ^*cas003*-NLS^ plasmids were transfected into HeLa cells. Mouse anti-FLAG primary antibody (1:1000; Genomics Technology) and goat-Alexa Fluore488-conjugated anti-mouse secondary antibody (1:500) were used for immunostaining. DAPI (1: 500, Beyotime) was applied for nucleus staining.

### Protein extraction and immunoblotting analysis

To extract the protein from the cell line, the cells were homogenized directly with 2 X SDS sample buffer and boiled for 5 minutes at 95℃. To obtain fish protein from the CHT region, the tails of embryos including the CHT region were cut down, then ultrasonicated in RIPA lysis buffer (50mM Tris(pH7.4), 150mM NaCl, 1% NP-40, 0.5% sodium deoxycholate, 0.1% SDS). After centrifugation at 12000rpm for 15 minutes, the supernatant was mixed with 2XSDS sample buffer and boiled for 10 minutes. Cytoplasmic and nuclear extracts were prepared from the 3dpf embryos with Nuclear and Cytoplasmic Protein Extraction Kit (Beyotime) according to the manufacturer’s instruction. The immunoblotting was carried out as previously described [85], with rabbit anti-phospho-Chk1 (Ser345) (133D) antibody (Cell Signaling Technology), rabbit anti-γH2AX antibody, rabbit anti-zebrafish TopBP1 antibody (generated by 840–940 amino acid of zebrafish TopBP1 protein as antigen), mouse anti-GAPDH antibody (1D4) (Santa Cruz), mouse anti-alpha-tubulin antibody (Sigma) or rabbit anti-Histon3 (H3) antibody (Abcam).

### Imaging

Images of zebrafish immunofluorescence staining or live transgenic embryos were taken by Olympus FV1000 scanning confocal microscope. The embryos were mounted in 1% low-melt agarose in a self-made 35mm coverslip-bottom dish. The confocal images were captured with an UPLSAPO 20X or 60X objective. To obtain images of HeLa cells immunostaining, the slides were directly immersed in the PBS solution in a 10cm dish. The images were captured with an UPLSAPO 40X objective. The transient transgenesis embryos and embryos for bright field imaging were anesthetized with 0.03% Tricaine (Sigma-Aldrich), mounted in 3% methylcellulose and imaged using a Zeiss Axio Zoom. V16 microscope equipped with a Zeiss AxioCam MRm digital camera.

### Statistics analysis

Data were analyzed with the Graphpad Prism 5 software using the two-tailed Student’s t-test. The plot error values were calculated by standard error of the mean (SEM). All data in this study were repeated for at least twice.

## Supporting Information

S1 Fig The vascular system is normal in mutant^*cas003*^ embryos. (A, B) WISH results of *flk1* at 36hpf in sibling and mutant^*cas003*^ embryos. All the embryos (n = 47) show the same *flk1* expression pattern. Red arrows indicate the dorsal aorta (DA); blue arrows indicate the posterior cardinal vein (PCV). (C, D) WISH results of *ephrinB2* at 26hpf in sibling and mutant^*cas003*^ embryos. All the embryos (n = 36) show the same *ephrinB2* expression pattern. Arrows indicate the DA. The expression of *flk1* and *ephrinB2* in mutant^*cas003*^ embryos is comparable to that in siblings. (E-L) Live imaging of vascular plexus in the CHT region of sibling and mutant^*cas003*^ embryos within Tg(*fli1*: EGFP) background from 2dpf to 5dpf. The vascular niche of HSPCs is normal in mutant^*cas003*^. For each panel, at least 6 embryos were observed. Scale bars represent 50 μm.
(TIF)

S2 Fig The primitive hematopoiesis is undisturbed in mutant^*cas003*^. (A-L) WISH results showing normal expression of *scl*, *gata1*, *pu*.*1*, *lyz*, *l-plastin* and *mpx* at 22hpf in mutant^*cas003*^ embryos comparing to siblings. Total numbers of embryos with *scl* or *gata1* stain are 56 and 76, respectively. The penetrance of the indicated phenotype is shown in the bottom left of each panel in C-L. Blue arrows indicate the myeloid cells in the yolk sac; black arrows indicate the granulocytes in the posterior blood island (PBI). (M)Quantitative analysis of *pu*.*1* ^+^, *lyz* ^+^, *l-plastin* ^+^ and *mpx* ^+^ cell numbers showing no significant difference between sibling and mutant^*cas003*^ embryos at 22hpf. Error bars represent SEM. ns represents no significance.
(TIF)

S3 Fig The *topbp1* morphants can phenocopy mutant^*cas003*^ embryos in a dose-dependent manner. (A) Diagram of *topbp1* MO knockdown effect evaluation construct. EGFP coding region was fused in frame to the 3’ end of a DNA fragment (blue boxes) containing *topbp1* ATG MO targeting site (red line). This construct was *in vitro* transcripted, and then co-injected with mCherry mRNA (50pg) and *topbp1* MO (1pg) or control MO (1pg) into 1-cell stage embryos. (B) Fluorescence of the 9hpf embryos in the *topbp1* knockdown effect evaluation assay. *topbp1* MO (upper), instead of control MO (down), can knockdown the expression of EGFP without affecting mCherry fluorescence. Left column, bright field; middle column, EGFP; right column, mCherry. (C) Quantitation of 22hpf morphology of the wild-type embryos injected with a gradient dose of *topbp1* MO. Injection with more than 1.6pg *topbp1* MO can induce abnormal morphogenesis. (D) Quantitation of the *c-myb* WISH analysis of embryos injected with a gradient dose of *topbp1* MO at 3dpf. The *topbp1* morphants can phenocopy *topbp1* ^*cas003*^ mutants with 1.6–2 pg injection dosage without causing morphological defect.
(TIF)

S4 Fig The HSPC formation, primitive hematopoiesis and vascular morphogenesis are normal in *topbp1* morphants. (A-H’) Time-course analysis of *c-myb* expression in control and *topbp1* morphants (1.6pg MO) from 36hpf to 5dpf. In *topbp1* morphants, the *c-myb* expression is normal at 36hpf and 48hpf, but is decreased at 4dpf and 5dpf. The penetrance of the indicated phenotype is shown in the bottom left of each panel. (A’-H’) Enlarged detail of *c-myb* WISH analysis in the CHT region. (I-P) WISH analysis of *scl*, *gata1* and *pu*.*1* at 22hpf, or *flk1* at 3dpf in control and *topbp1* morphants (1.6pg MO). The primitive hematopoiesis and vascular system are normal in *topbp1* morphants. (Q-R) Live imaging analysis of vascular plexus in the CHT region in control or *topbp1* morphants within Tg(*fli1*: EGFP) background at 3dpf. The vascular plexus is normal in *topbp1* morphants. Scale bars represent 50μm.
(TIF)

S5 Fig The *topbp1* gene is ubiquitously expressed in the development. (A-J) WISH results of *topbp1* from 1-cell stage to 5dpf showing global expression of *topbp1*. ss, somites. The penetrance of the indicated phenotype is shown in the bottom left of each panel. (K) Quantitation of *topbp1* in the whole embryos, tails and sorted CD41^+^ cells at the indicated stage. *topbp1* is 3-fold enriched in CD41^+^ cells within the tail region of Tg(*CD41*: EGFP) line at 5dpf, demonstrating the expression of *topbp1* in HSPCs. *c-myb* is used as a positive control. (L) Western blotting analysis on endogenous TopBP1^*WT*^/TopBP1^*cas003*^ protein in cytoplasmic and nuclear fractions of pooled 3dpf embryos from heterozygotes incrossing. TopBP1^*WT*^ localized in nucleus, but TopBP1^*cas003*^ localized in cytosol. (M-P) WISH analysis of *topbp1* in sibling and *topbp1* ^*cas003*^ mutant embryos at 3dpf. The expression of *topbp1* is decreased in mutant, especially in cranial region. (N, P) Enlarged detail of *c-myb* WISH analysis in CHT region. (Q) Quantitative PCR analysis on the *topbp1* mRNA level in the whole embryos at 5dpf or the tails including CHT from 2dpf to 5dpf. The expression level of *topbp1* is decreased in the *topbp1* ^*cas003*^ mutants. Error bars represent SEM; * represents *p*<0.05; *** represents *p*<0.001.
(TIF)

S6 Fig Bcl2 overexpression can rescue hematopoietic failure in *topbp1* ^*cas003*^ mutants. (A) Quantitative analysis of HSPCs phenotype, monitored by *c-myb* WISH, in *topbp1* ^*cas003*^ mutants with or without *bcl2* mRNA injection. *bcl2* mRNA could significantly rescue *c-myb* expression in *topbp1* ^*cas003*^ mutants. The number of the mutant embryos (n) is indicated above each column. (B-D’) WISH of *c-myb* in sibling, *topbp1* ^*cas003*^ mutants and mutants injected with *bcl2* mRNA at 4dpf. The proportion of the rescued *c-myb* phenotype shown in D is 25 out of 43 mutant embryos. (B’-D’) Enlarged views of the CHT representing the dashed boxes region in the left column.
(TIF)

S7 Fig Conserved protein-protein interaction region among vertebrate TopBP1. In zebrafish TopBP1 (Dr. TopBP1), R122, R669 and W1156 sites are essential for the TopBP1 interaction with Rad9, MDC1 and ATR activation, respectively. The positions of these 3 sites are shown in the schematic diagram. Alignments of these sites among zebrafish, mice and human are shown in the bottom. All these sites are highly conserved.
(TIF)

S8 Fig DNA damage is accumulated in HSPCs in the CHT region of *topbp1* ^*cas003*^ mutants. (A-B) Triple staining of γH2AX antibody, *c-myb* fluorescent *in situ* hybridization and DAPI in *topbp1* ^*cas003*^ mutants and siblings at 39hpf. The triple staining results show that the γH2AX^+^ HSPCs, which are undetectable in the AGM region in both mutants and siblings (A), are increased in the CHT region of *topbp1* ^*cas003*^ mutants at 39hpf (B). The right columns in B are the magnified views of the dashed boxes in the middle columns. Scale bars represent 50um. (C) Quantification of γH2AX^+^ HSPCs in the AGM or CHT region in *topbp1* ^*cas003*^ mutants and siblings at 39hpf, 2dpf and 3.5dpf. The number of γH2AX^+^ HSPCs is increased in the CHT region in the mutants from 39hpf to 3.5dpf. Error bars represent SEM. ns, no significance; *, *p*<0.05; ***, *p*<0.001.
(TIF)

S9 Fig The *topbp1* ^*cas003*^ mutants are sensitive to the HU treatment. (A) The procedure of hydroxyurea (HU) treatment. (B) Quantitative analysis of embryonic lethality of wild-type siblings and *topbp1* ^*cas003*^ mutants after 250mM HU treatment as indicated in A. The numbers of embryos are shown above the columns. More *topbp1* ^*cas003*^ mutants are lethal after HU treatment.
(TIF)

S10 Fig Biallelic disruption of *topbp1* by CRISP/Cas9 mimics HSPCs deficiency in topbp1^*cas003*^ mutant. (A) Diagram showing the target site of *topbp1* gRNA used in this study. The *topbp1* gene contains 27 exons. Five gRNAs targeting exon 6, 7 and 9 of *topbp1* were designed. The indicated gRNA targeting exon 9 with high mutagenesis rates was used for the following assay. Arrow indicates the target site of the gRNA. Red asterisk represents the position of nonsense mutation in *topbp1* ^*cas003*^ mutant. (B) T7 endonuclease I (T7EI) assay showing the mutagenesis efficacy in *topbp1* gRNA targeted embryos. *topbp1* gRNA (25pg) and *nls*-zCas9-*nls* mRNA (150pg) were injected into the wild-type embryos. M, maker; Emb, embryo. (C-D’) The *c-myb* WISH results showing some of the Cas9 injected wild-type embryos manifested dramatically decreased *c-myb* expression as same as *topbp1* ^*cas003*^ mutant at 4dpf (4/53). (E-F’) The *c-myb* WISH results showing the Cas9 injected WT/Het embryos displayed dramatically decreased *c-myb* expression at 4dpf (14/55). Het, *topbp1* ^*cas003*^ heterozygote. WT/Het embryos were generated from outcross of *topbp1* ^*cas003*^ heterozygote and wild-type fish (Efficiency of CRISPR/Cas9-mediated mutagenesis varies in different microinjection assay). (G-H) Genomic sequencing of the *topbp1* gRNA targeting region in the WT or WT/Het embryos (G) and Cas9 injected embryos in D or F (H). Red boxes represent the protospacer-adjacent motif (PAM) site; red arrows indicate the orientation and target site of *topbp1* gRNA; blue arrows show the orientation of sequencing. (I) Mutations in 7 out of 9 sequenced *topbp1* alleles from a *topbp1*-targeted F0 embryo. The wild-type reference sequence is underlined. The target site is showed in blue; PAM is highlighted by yellow background. Deletions and insertions are indicated by dashes and lowercase red letters, respectively. The indel mutations are noted at the right of each sequence (+, insertion; −, deletion).
(TIF)

S11 Fig *lmo2* promoter-induced ectopic expression of wild-type *topbp1* can partially rescue HSPCs defect in *topbp1* ^*cas003*^ mutant. (A) Fluorescence observation of embryos injected with *lmo2*: *topbp1* ^WT^ transient transgenesis construct. P2A-mCherry fragment was added after *topbp1* ^WT^ as an indicator. The mCherry positive cells could be detected in the CHT region at 5dpf in the injected embryos instead of non-injected embryos. Arrow heads represent the melanocytes; arrows indicate the mCherry+ cells. (B-E’) The *c-myb* WISH analysis of *topbp1* ^*cas003*^ mutant embryos with ectopic expression of *ubi*: *topbp1WT* or *lmo2*: *topbp1* ^WT^. Ectopic expression of *lmo2*: *topbp1* ^WT^could partially rescue the *c-myb* expression in *topbp1* ^*cas003*^ mutants at 5dpf. (B) Quantitation of the rescue assay. (C’-E’) Enlarged views of the CHT regions in the left column.
(TIF)

S12 Fig Ectopic expression of TopBP1^*cas003*^ does not cause hematopoietic failure. The *c-myb* WISH analysis of wild-type embryos injected with *ubi*: *topbp1* ^*cas003*^ or control transient transgenesis constructs, showing no difference in definitive hematopoiesis at 5dpf.
(TIF)

S13 Fig The HSPCs undergo continuous propagation in the CHT region from 2dpf to 5dpf. Quantification of the percentage of BrdU^+^ HSPCs in the CHT region and the percentage of BrdU^+^ neural tube cells from 2dpf to 5dpf. The HSPCs are in a constant state of proliferation, although the propagation of neural tube cells is gradually decreased from 2dpf to 5dpf.
(TIF)

S1 Table Summarized information of oligos applied in this study. (PDF)

S1 Movie Bright field observation of wild-type sibling at 5dpf. (MOV)

S2 Movie Bright field observation of mutant^*cas003*^ at 5dpf. The heart beat and circulation are not disturbed in the mutant^*cas003*^ comparing to the wild-type sibling at 5dpf.
(MOV)
