# Supplementary figures and images for: TopBP1 Governs Hematopoietic Stem/Progenitor Cells Survival in Zebrafish Definitive Hematopoiesis
Source: PLoS Genet. 2015 Jul 1;11(7):e1005346. doi: 10.1371/journal.pgen.1005346 (PMC4488437; doi:10.1371/journal.pgen.1005346)

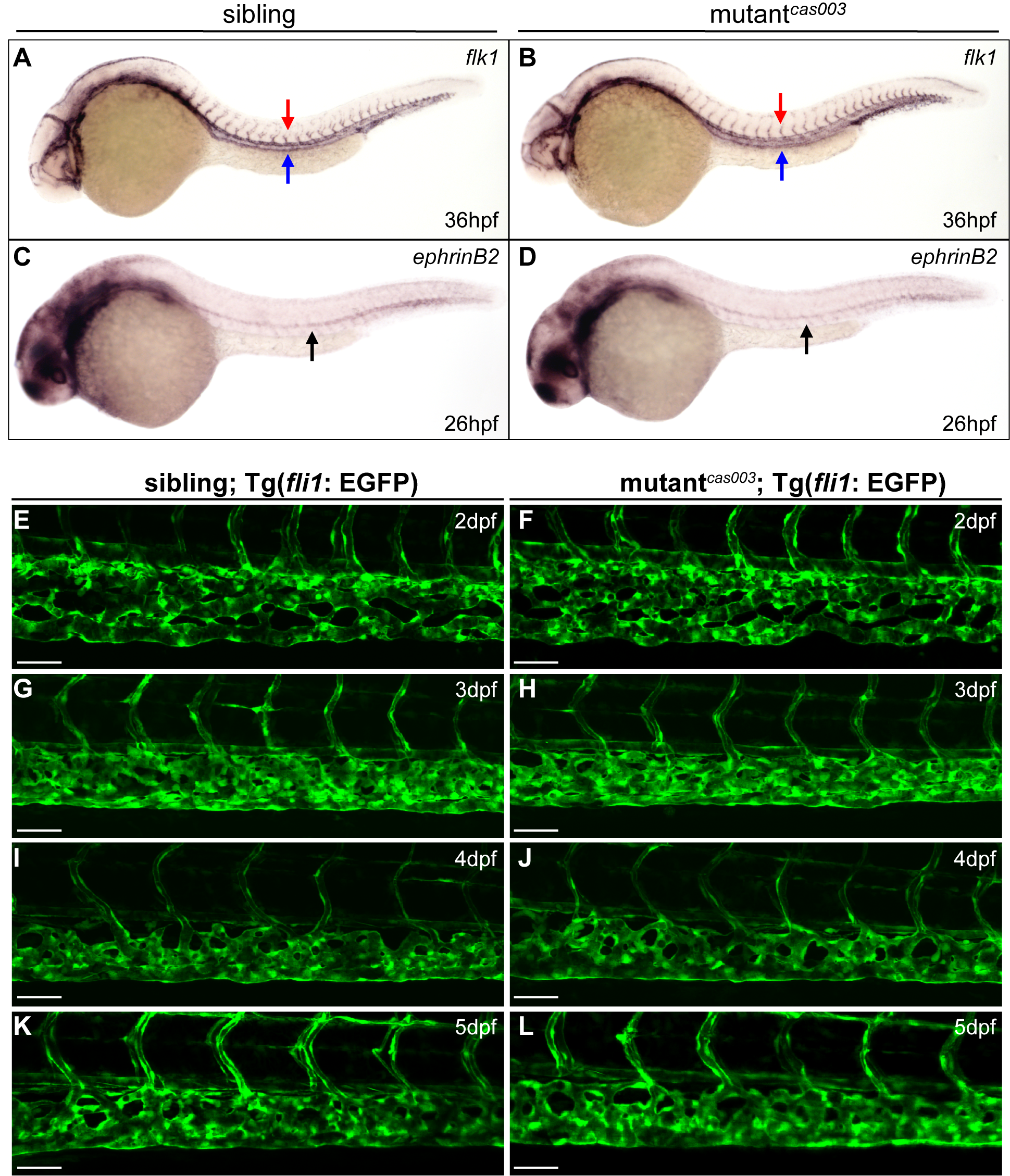

Supplement: S1 Fig — (A, B) WISH results of flk1 at 36hpf in sibling and mutantcas003 embryos. All the embryos (n = 47) show the same flk1 expression pattern. Red arrows indicate the dorsal aorta (DA); blue arrows indicate the posterior cardinal vein (PCV). (C, D) WISH results of ephrinB2 at 26hpf in sibling and mutantcas003 embryos. All the embryos (n = 36) show the same ephrinB2 expression pattern. Arrows indicate the DA. The expression of flk1 and ephrinB2 in mutantcas003 embryos is comparable to that in siblings. (E-L) Live imaging of vascular plexus in the CHT region of sibling and mutantcas003 embryos within Tg(fli1: EGFP) background from 2dpf to 5dpf. The vascular niche of HSPCs is normal in mutantcas003. For each panel, at least 6 embryos were observed. Scale bars represent 50 μm. (TIF) [file pgen.1005346.s001.tif]

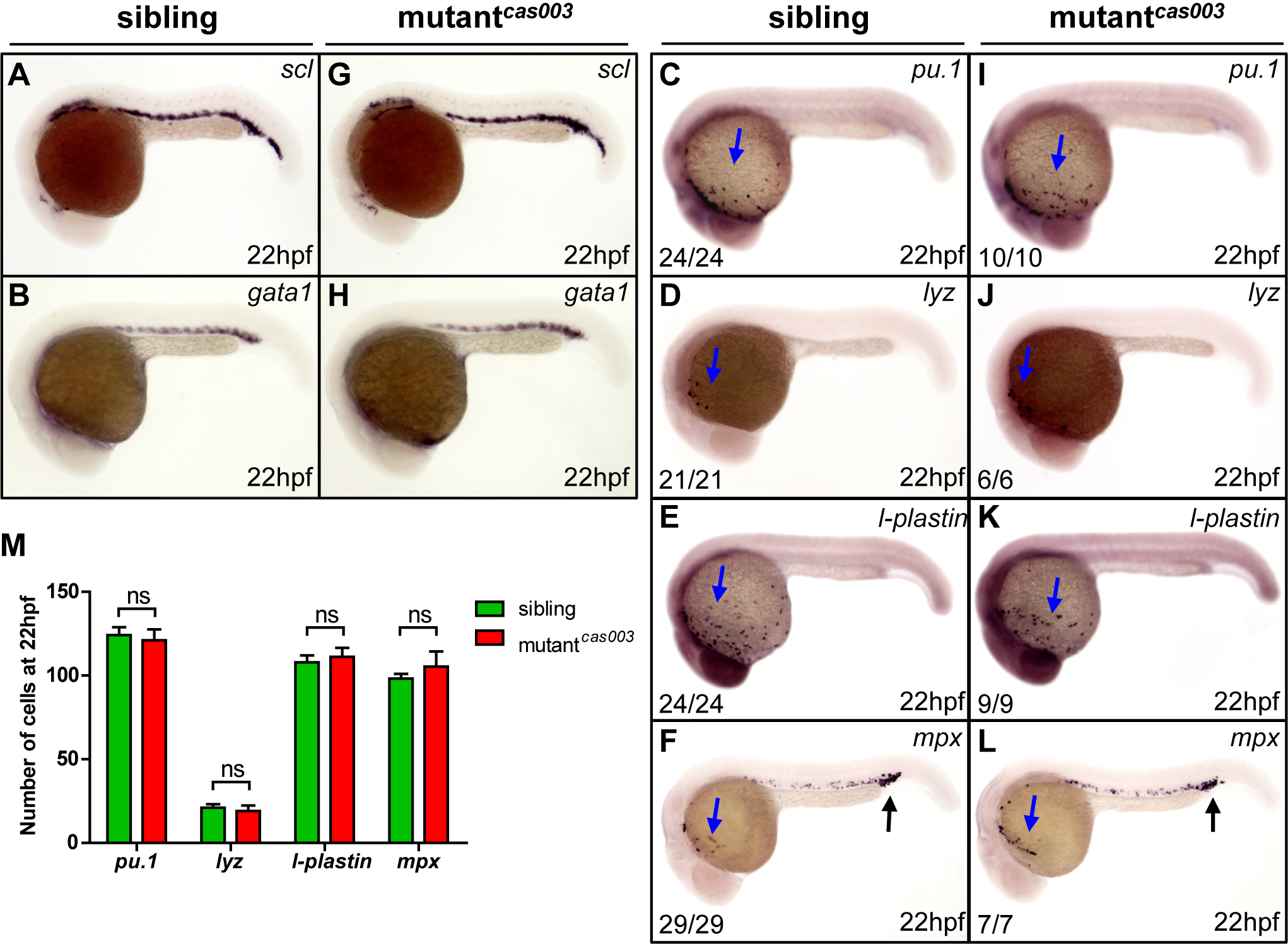

Supplement: S2 Fig — (A-L) WISH results showing normal expression of scl, gata1, pu.1, lyz, l-plastin and mpx at 22hpf in mutantcas003 embryos comparing to siblings. Total numbers of embryos with scl or gata1 stain are 56 and 76, respectively. The penetrance of the indicated phenotype is shown in the bottom left of each panel in C-L. Blue arrows indicate the myeloid cells in the yolk sac; black arrows indicate the granulocytes in the posterior blood island (PBI). (M)Quantitative analysis of pu.1 +, lyz +, l-plastin + and mpx + cell numbers showing no significant difference between sibling and mutantcas003 embryos at 22hpf. Error bars represent SEM. ns represents no significance. (TIF) [file pgen.1005346.s002.tif]

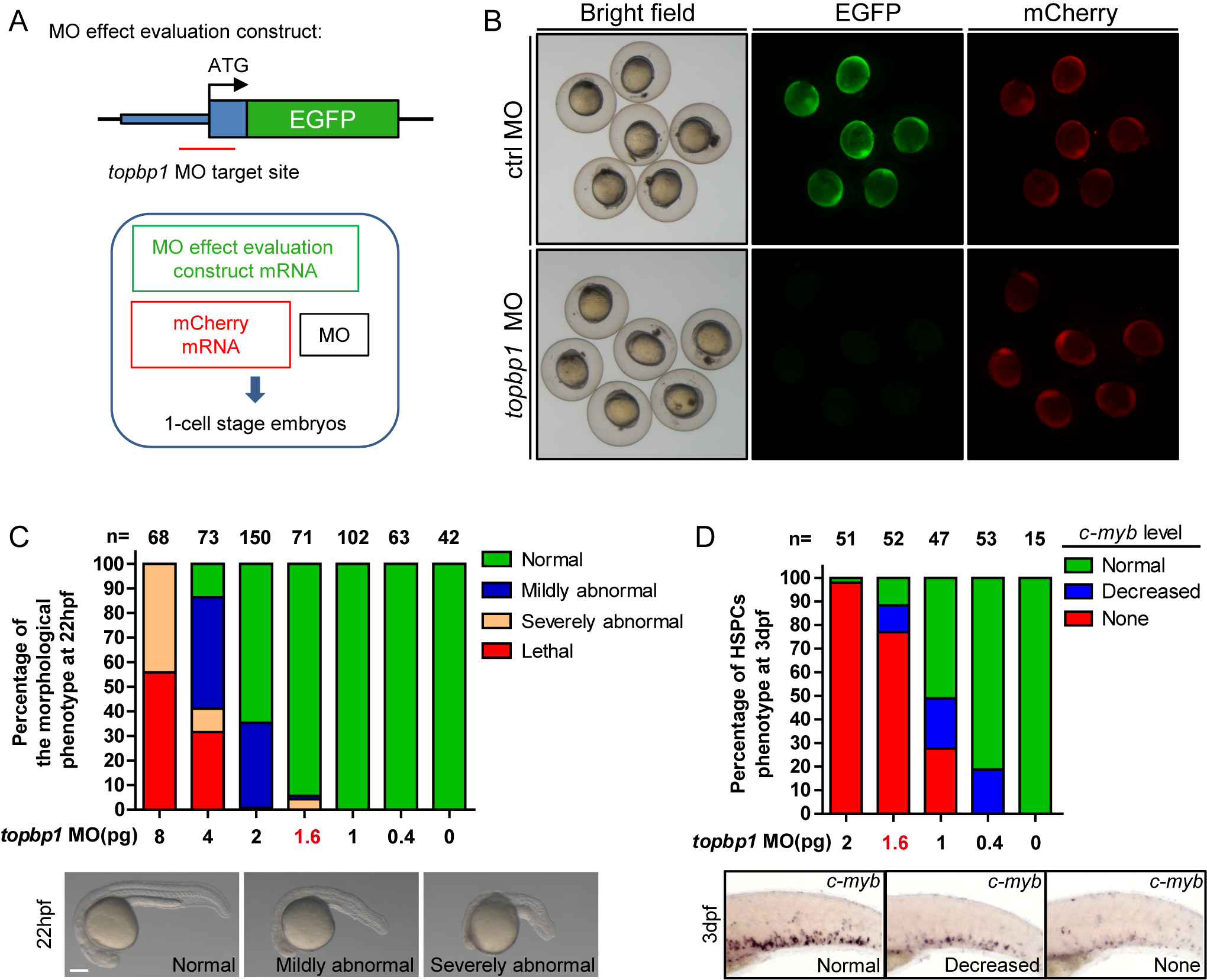

Supplement: S3 Fig — (A) Diagram of topbp1 MO knockdown effect evaluation construct. EGFP coding region was fused in frame to the 3’ end of a DNA fragment (blue boxes) containing topbp1 ATG MO targeting site (red line). This construct was in vitro transcripted, and then co-injected with mCherry mRNA (50pg) and topbp1 MO (1pg) or control MO (1pg) into 1-cell stage embryos. (B) Fluorescence of the 9hpf embryos in the topbp1 knockdown effect evaluation assay. topbp1 MO (upper), instead of control MO (down), can knockdown the expression of EGFP without affecting mCherry fluorescence. Left column, bright field; middle column, EGFP; right column, mCherry. (C) Quantitation of 22hpf morphology of the wild-type embryos injected with a gradient dose of topbp1 MO. Injection with more than 1.6pg topbp1 MO can induce abnormal morphogenesis. (D) Quantitation of the c-myb WISH analysis of embryos injected with a gradient dose of topbp1 MO at 3dpf. The topbp1 morphants can phenocopy topbp1 cas003 mutants with 1.6–2 pg injection dosage without causing morphological defect. (TIF) [file pgen.1005346.s003.tif]

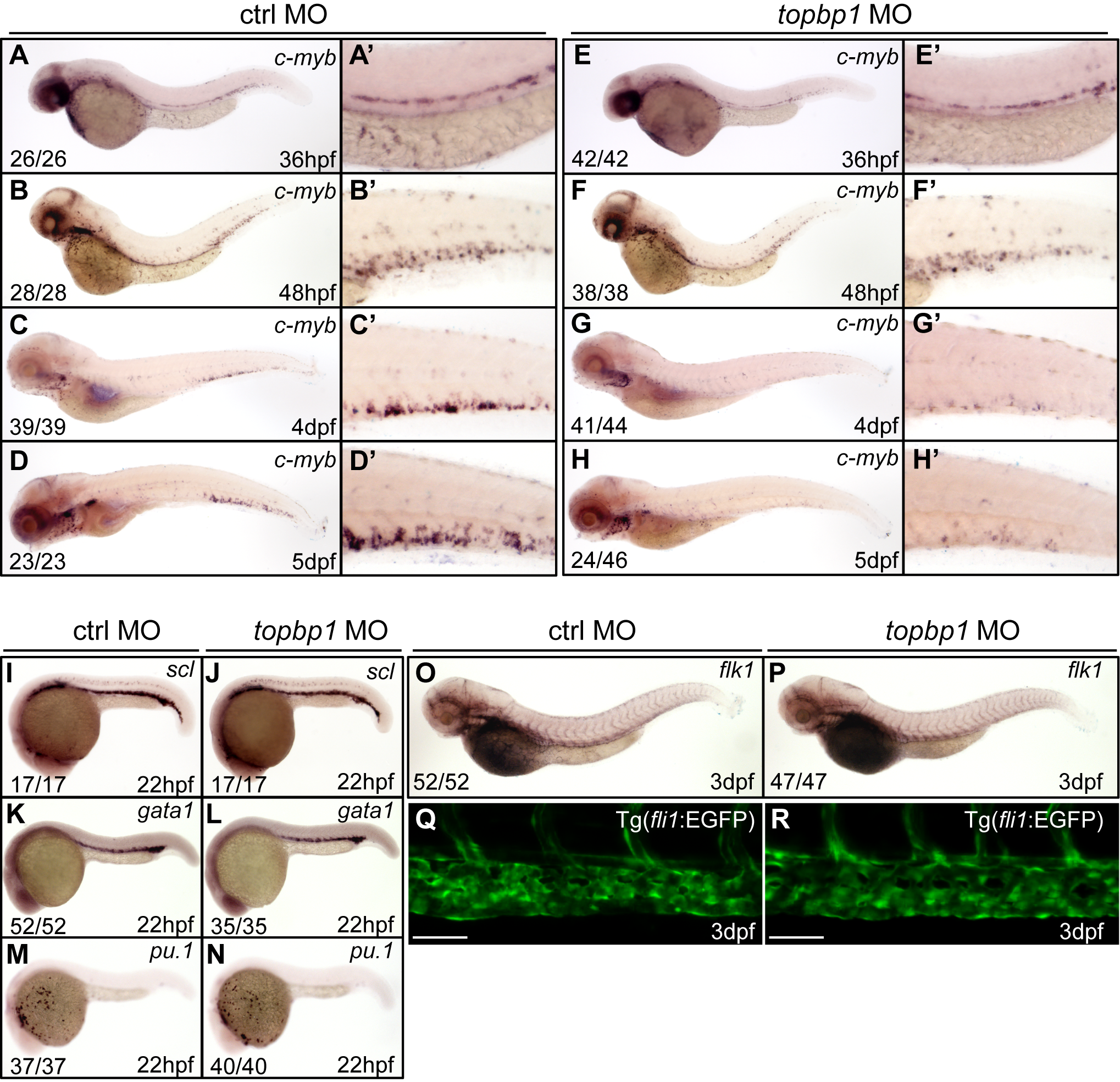

Supplement: S4 Fig — (A-H’) Time-course analysis of c-myb expression in control and topbp1 morphants (1.6pg MO) from 36hpf to 5dpf. In topbp1 morphants, the c-myb expression is normal at 36hpf and 48hpf, but is decreased at 4dpf and 5dpf. The penetrance of the indicated phenotype is shown in the bottom left of each panel. (A’-H’) Enlarged detail of c-myb WISH analysis in the CHT region. (I-P) WISH analysis of scl, gata1 and pu.1 at 22hpf, or flk1 at 3dpf in control and topbp1 morphants (1.6pg MO). The primitive hematopoiesis and vascular system are normal in topbp1 morphants. (Q-R) Live imaging analysis of vascular plexus in the CHT region in control or topbp1 morphants within Tg(fli1: EGFP) background at 3dpf. The vascular plexus is normal in topbp1 morphants. Scale bars represent 50μm. (TIF) [file pgen.1005346.s004.tif]

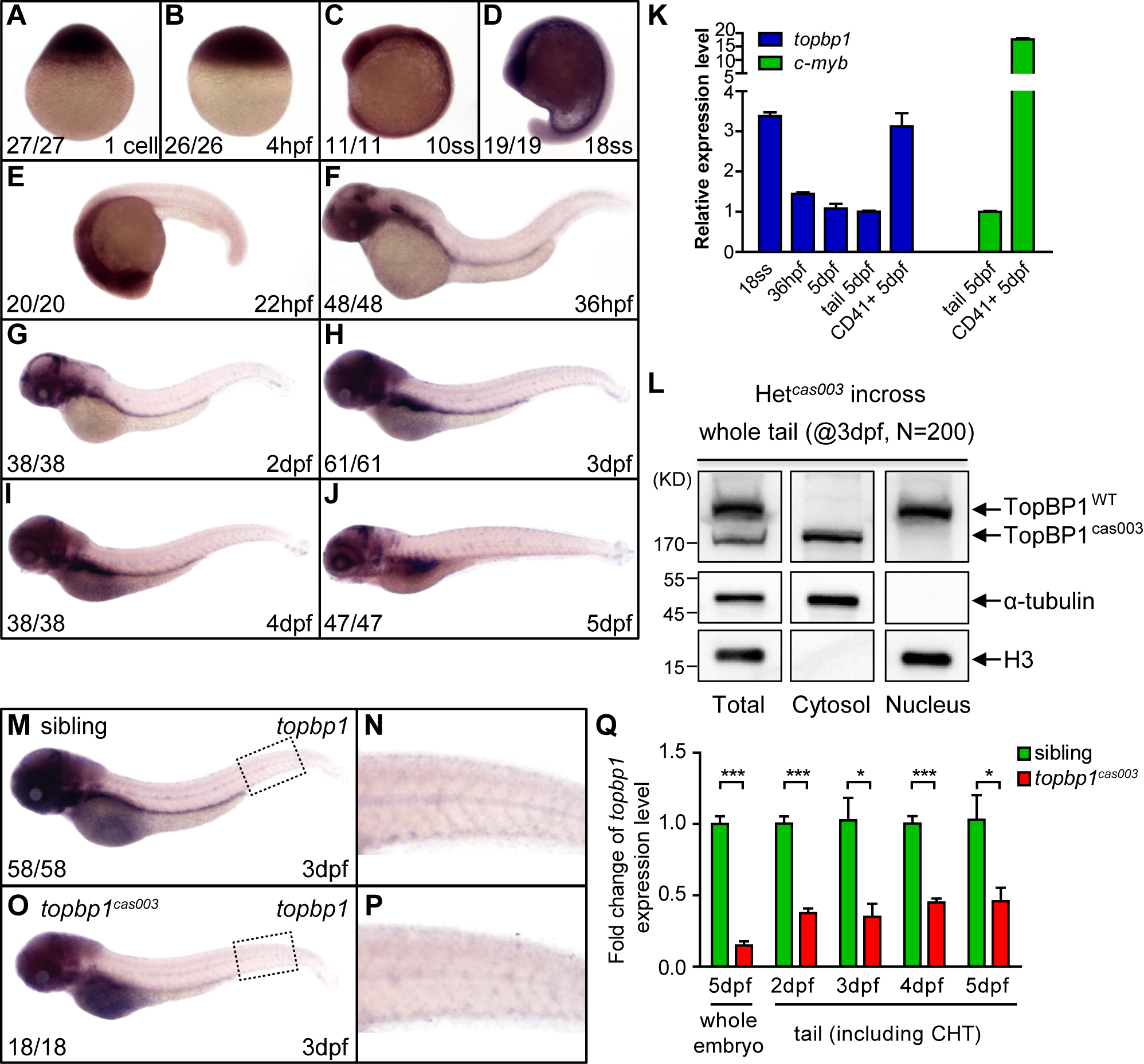

Supplement: S5 Fig — (A-J) WISH results of topbp1 from 1-cell stage to 5dpf showing global expression of topbp1. ss, somites. The penetrance of the indicated phenotype is shown in the bottom left of each panel. (K) Quantitation of topbp1 in the whole embryos, tails and sorted CD41+ cells at the indicated stage. topbp1 is 3-fold enriched in CD41+ cells within the tail region of Tg(CD41: EGFP) line at 5dpf, demonstrating the expression of topbp1 in HSPCs. c-myb is used as a positive control. (L) Western blotting analysis on endogenous TopBP1WT/TopBP1cas003 protein in cytoplasmic and nuclear fractions of pooled 3dpf embryos from heterozygotes incrossing. TopBP1WT localized in nucleus, but TopBP1cas003 localized in cytosol. (M-P) WISH analysis of topbp1 in sibling and topbp1 cas003 mutant embryos at 3dpf. The expression of topbp1 is decreased in mutant, especially in cranial region. (N, P) Enlarged detail of c-myb WISH analysis in CHT region. (Q) Quantitative PCR analysis on the topbp1 mRNA level in the whole embryos at 5dpf or the tails including CHT from 2dpf to 5dpf. The expression level of topbp1 is decreased in the topbp1 cas003 mutants. Error bars represent SEM; * represents p<0.05; *** represents p<0.001. (TIF) [file pgen.1005346.s005.tif]

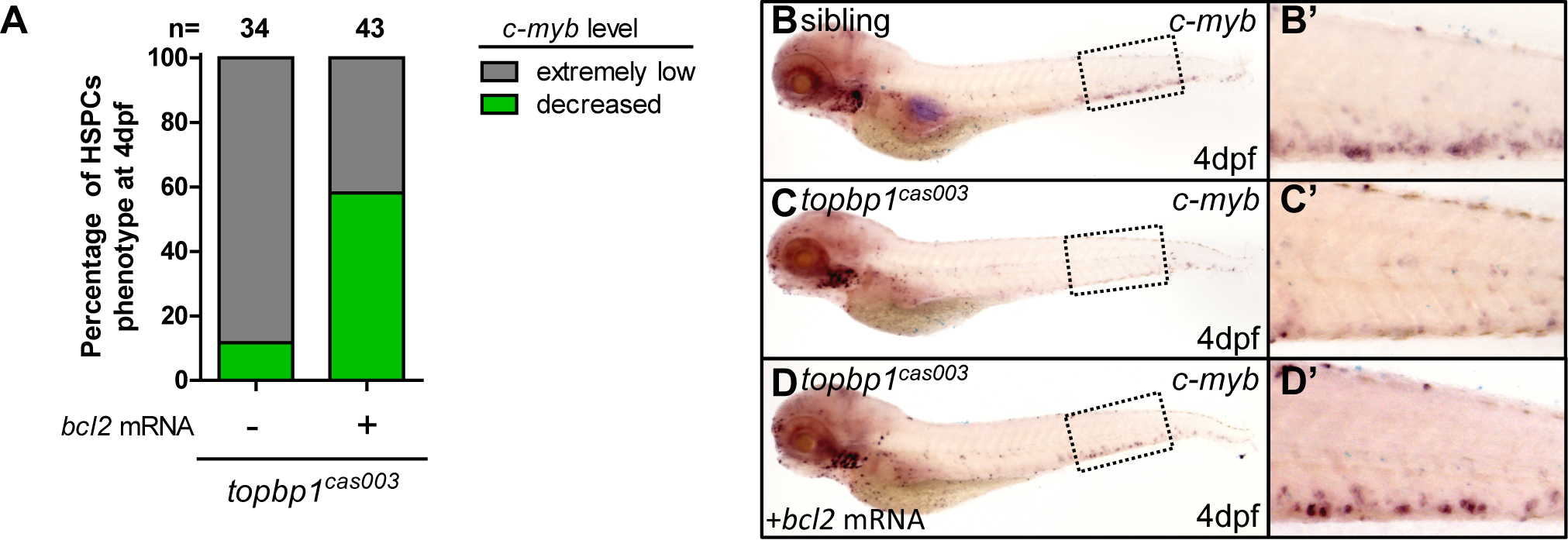

Supplement: S6 Fig — (A) Quantitative analysis of HSPCs phenotype, monitored by c-myb WISH, in topbp1 cas003 mutants with or without bcl2 mRNA injection. bcl2 mRNA could significantly rescue c-myb expression in topbp1 cas003 mutants. The number of the mutant embryos (n) is indicated above each column. (B-D’) WISH of c-myb in sibling, topbp1 cas003 mutants and mutants injected with bcl2 mRNA at 4dpf. The proportion of the rescued c-myb phenotype shown in D is 25 out of 43 mutant embryos. (B’-D’) Enlarged views of the CHT representing the dashed boxes region in the left column. (TIF) [file pgen.1005346.s006.tif]

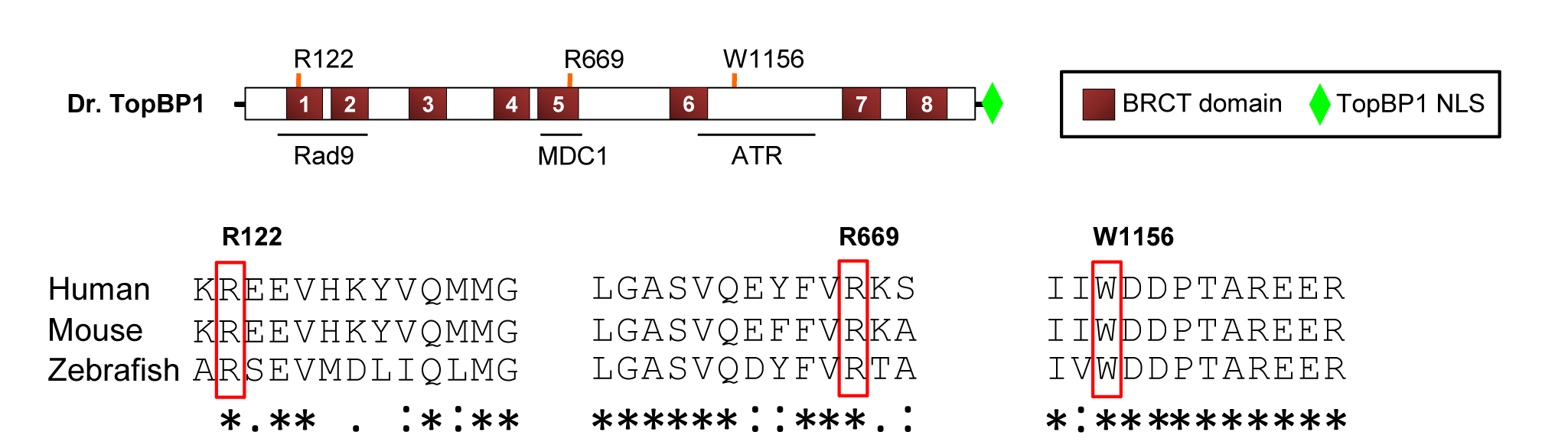

Supplement: S7 Fig — In zebrafish TopBP1 (Dr. TopBP1), R122, R669 and W1156 sites are essential for the TopBP1 interaction with Rad9, MDC1 and ATR activation, respectively. The positions of these 3 sites are shown in the schematic diagram. Alignments of these sites among zebrafish, mice and human are shown in the bottom. All these sites are highly conserved. (TIF) [file pgen.1005346.s007.tif]

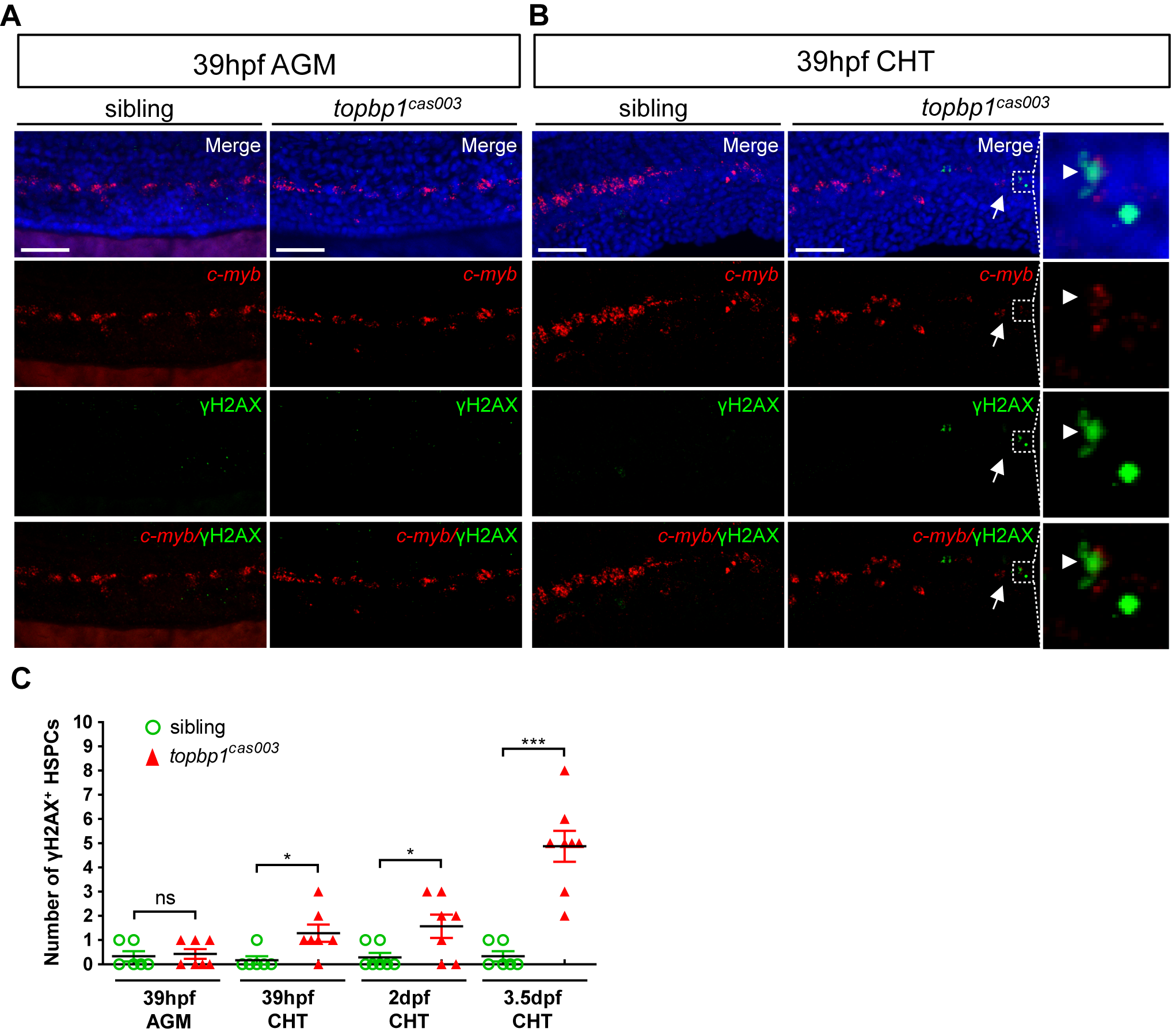

Supplement: S8 Fig — (A-B) Triple staining of γH2AX antibody, c-myb fluorescent in situ hybridization and DAPI in topbp1 cas003 mutants and siblings at 39hpf. The triple staining results show that the γH2AX+ HSPCs, which are undetectable in the AGM region in both mutants and siblings (A), are increased in the CHT region of topbp1 cas003 mutants at 39hpf (B). The right columns in B are the magnified views of the dashed boxes in the middle columns. Scale bars represent 50um. (C) Quantification of γH2AX+ HSPCs in the AGM or CHT region in topbp1 cas003 mutants and siblings at 39hpf, 2dpf and 3.5dpf. The number of γH2AX+ HSPCs is increased in the CHT region in the mutants from 39hpf to 3.5dpf. Error bars represent SEM. ns, no significance; *, p<0.05; ***, p<0.001. (TIF) [file pgen.1005346.s008.tif]

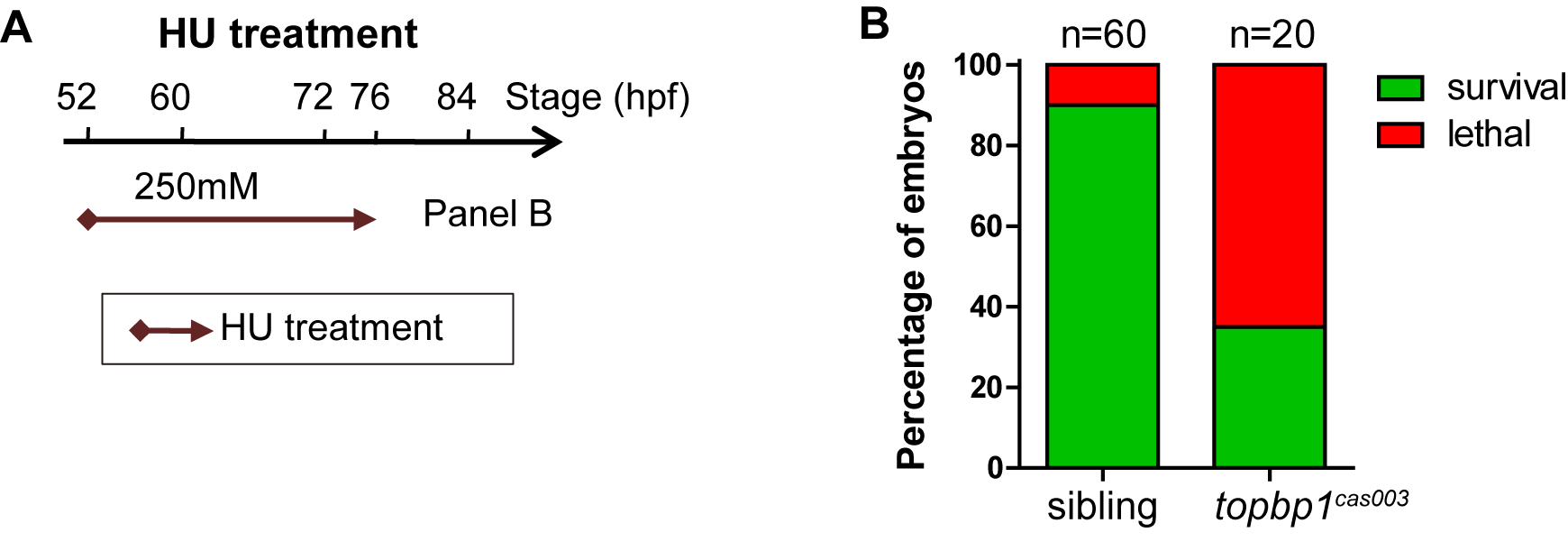

Supplement: S9 Fig — (A) The procedure of hydroxyurea (HU) treatment. (B) Quantitative analysis of embryonic lethality of wild-type siblings and topbp1 cas003 mutants after 250mM HU treatment as indicated in A. The numbers of embryos are shown above the columns. More topbp1 cas003 mutants are lethal after HU treatment. (TIF) [file pgen.1005346.s009.tif]

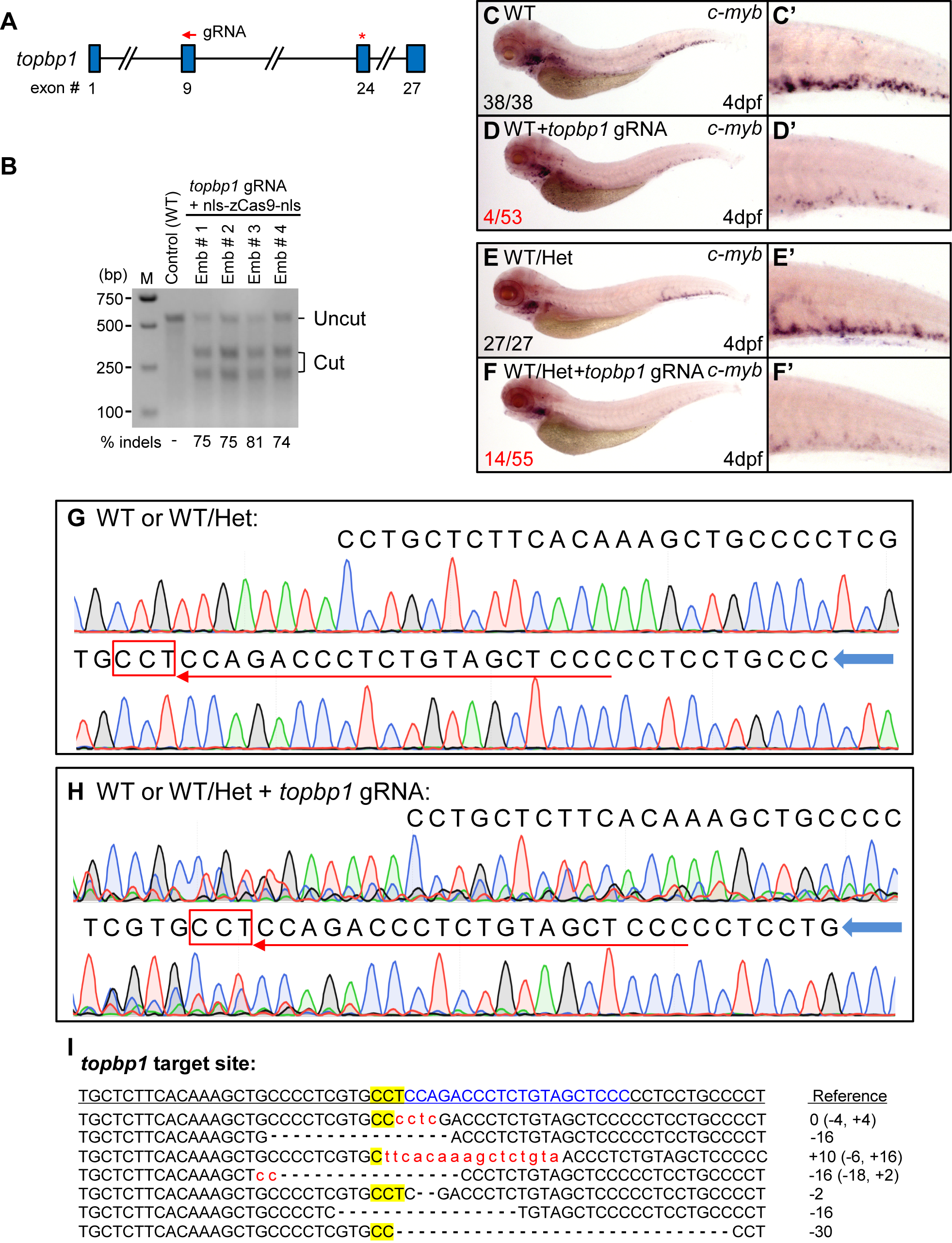

Supplement: S10 Fig — (A) Diagram showing the target site of topbp1 gRNA used in this study. The topbp1 gene contains 27 exons. Five gRNAs targeting exon 6, 7 and 9 of topbp1 were designed. The indicated gRNA targeting exon 9 with high mutagenesis rates was used for the following assay. Arrow indicates the target site of the gRNA. Red asterisk represents the position of nonsense mutation in topbp1 cas003 mutant. (B) T7 endonuclease I (T7EI) assay showing the mutagenesis efficacy in topbp1 gRNA targeted embryos. topbp1 gRNA (25pg) and nls-zCas9-nls mRNA (150pg) were injected into the wild-type embryos. M, maker; Emb, embryo. (C-D’) The c-myb WISH results showing some of the Cas9 injected wild-type embryos manifested dramatically decreased c-myb expression as same as topbp1 cas003 mutant at 4dpf (4/53). (E-F’) The c-myb WISH results showing the Cas9 injected WT/Het embryos displayed dramatically decreased c-myb expression at 4dpf (14/55). Het, topbp1 cas003 heterozygote. WT/Het embryos were generated from outcross of topbp1 cas003 heterozygote and wild-type fish (Efficiency of CRISPR/Cas9-mediated mutagenesis varies in different microinjection assay). (G-H) Genomic sequencing of the topbp1 gRNA targeting region in the WT or WT/Het embryos (G) and Cas9 injected embryos in D or F (H). Red boxes represent the protospacer-adjacent motif (PAM) site; red arrows indicate the orientation and target site of topbp1 gRNA; blue arrows show the orientation of sequencing. (I) Mutations in 7 out of 9 sequenced topbp1 alleles from a topbp1-targeted F0 embryo. The wild-type reference sequence is underlined. The target site is showed in blue; PAM is highlighted by yellow background. Deletions and insertions are indicated by dashes and lowercase red letters, respectively. The indel mutations are noted at the right of each sequence (+, insertion; −, deletion). (TIF) [file pgen.1005346.s010.tif]

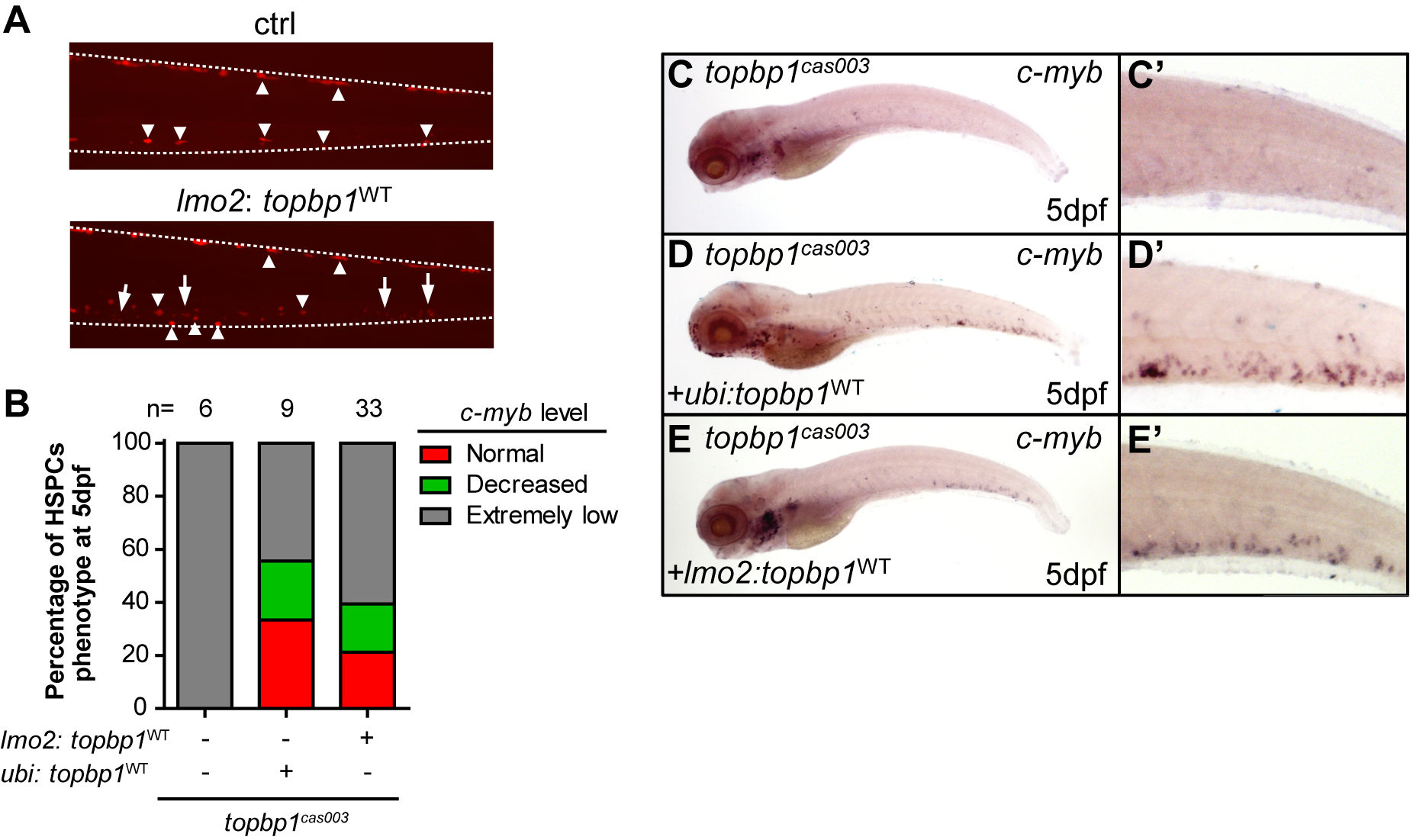

Supplement: S11 Fig — (A) Fluorescence observation of embryos injected with lmo2: topbp1 WT transient transgenesis construct. P2A-mCherry fragment was added after topbp1 WT as an indicator. The mCherry positive cells could be detected in the CHT region at 5dpf in the injected embryos instead of non-injected embryos. Arrow heads represent the melanocytes; arrows indicate the mCherry+ cells. (B-E’) The c-myb WISH analysis of topbp1 cas003 mutant embryos with ectopic expression of ubi: topbp1WT or lmo2: topbp1 WT. Ectopic expression of lmo2: topbp1 WTcould partially rescue the c-myb expression in topbp1 cas003 mutants at 5dpf. (B) Quantitation of the rescue assay. (C’-E’) Enlarged views of the CHT regions in the left column. (TIF) [file pgen.1005346.s011.tif]

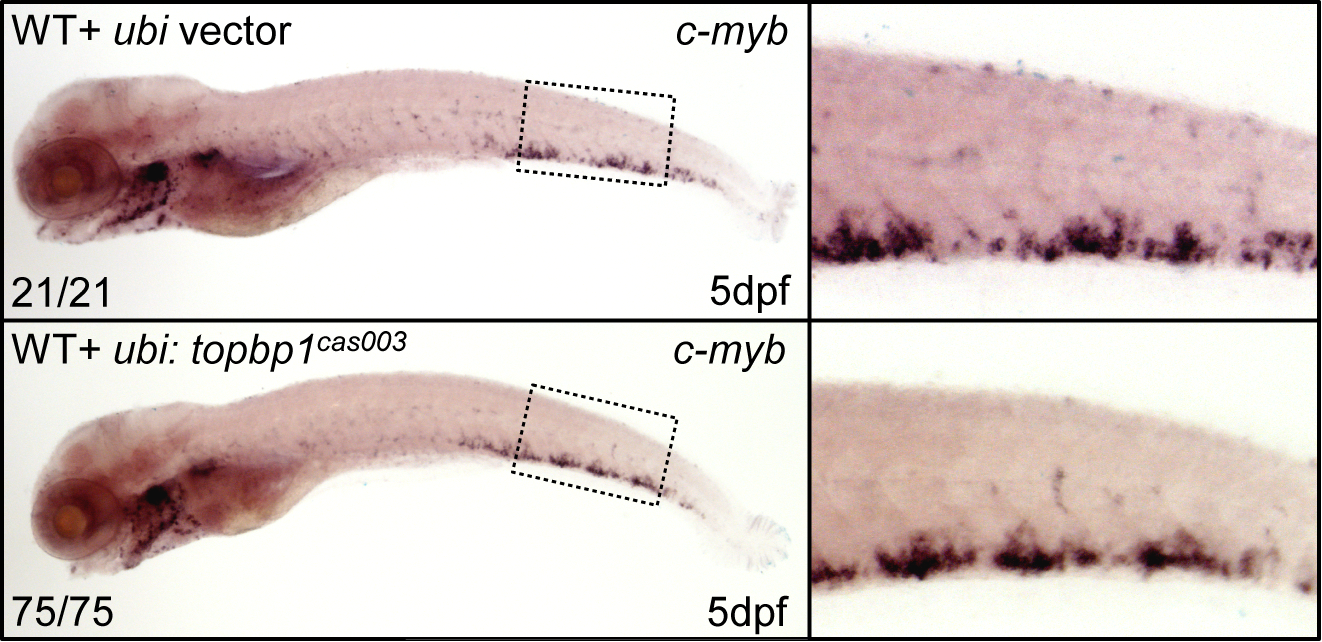

Supplement: S12 Fig — The c-myb WISH analysis of wild-type embryos injected with ubi: topbp1 cas003 or control transient transgenesis constructs, showing no difference in definitive hematopoiesis at 5dpf. (TIF) [file pgen.1005346.s012.tif]

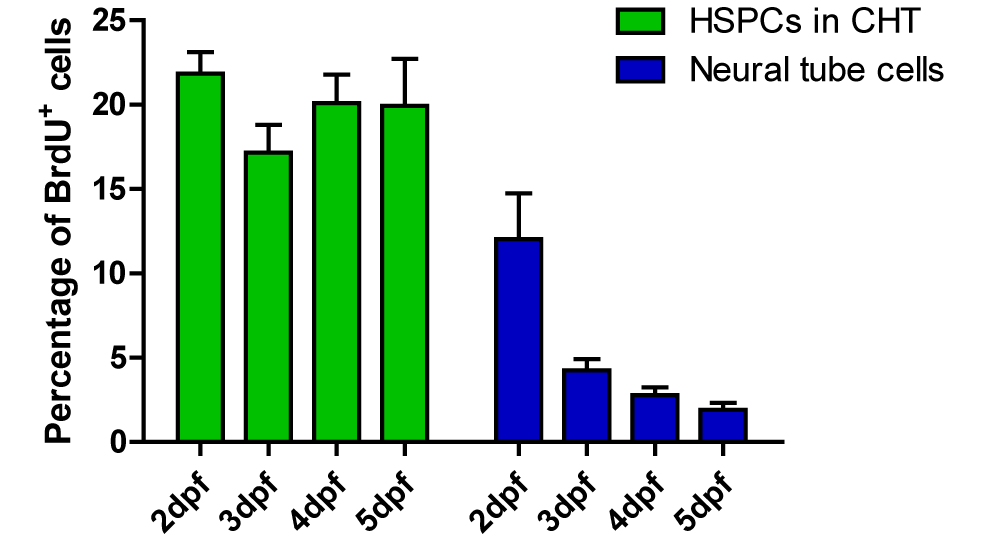

Supplement: S13 Fig — Quantification of the percentage of BrdU+ HSPCs in the CHT region and the percentage of BrdU+ neural tube cells from 2dpf to 5dpf. The HSPCs are in a constant state of proliferation, although the propagation of neural tube cells is gradually decreased from 2dpf to 5dpf. (TIF) [file pgen.1005346.s013.tif]
