# Supplementary material for: TopBP1 Governs Hematopoietic Stem/Progenitor Cells Survival in Zebrafish Definitive Hematopoiesis
Source: PLoS Genet. 2015 Jul 1;11(7):e1005346. doi: 10.1371/journal.pgen.1005346 (PMC4488437; doi:10.1371/journal.pgen.1005346)
Supplement: S1 Table — (PDF) [file pgen.1005346.s014.pdf]

1. Primers of SSLP markers

SSLP markers	Forward primer	Reverse primer
z9852	ACAGCTCTTCTCCAGCAGGA	GTGCCTTCCAATAAAGCCAA
zC285f17_10	GGAACAGTAGATCCGATTTGG	CGCTTGCTGTTTTAATAGACTGG
L0310_5	GCACCGAGAAGAAAAGCAAG	GAGATCTGAGGGAGGCACAA
R0310_4	GTCCAGCCTTCACAGTCCTC	CCACAACTTTCACCTGCTGT
zKp105G12_4	TCTGGGCCATATGGGAGTTA	AGCAGAGTCTGGCTAGAGTGG
R0306_4	CTCAGCTGCCCTCTCTATGG	ATTCAGCATCACAGCAGCAG

2. Primers for *topbp1* cDNA sequencing

Name	Forward primer	Reverse primer
topbp1 _1	CATGTCACCATGTCGAAAGC	CCTGTGTAGGTGGCTCCATT
topbp1 _2	AGCATGCTGGGAAAAGTCTC	AAGGAACCACGGGAAAAACT
topbp1 _3	CTGGAGGTCTGCGCTTTAAC	TTGGCGAGCTGAATAAGTGA
topbp1 _4	GTGGAGCAGCAGTGTGTGTT	CGAGGTTTGTGACGTGACTG
topbp1 _5	TCGGACCGTCATTTAACACA	TGGCAGACATGATCTCCTGA
topbp1 _6	AGTGTGGGAGAGCCTGAGAA	ATCACATGAGTGCAGCTTGG
topbp1 _7	AGAAGGATCCTCCACGGTTT	AATTTGAGCTTGAAATAGGATGA

3. Primers for *topbp1* genomic DNA sequencing

Name	Forward primer	Reverse primer
topbp1 -gp1	ATCAGTACGAGTGGGGAAGC	CCTCTCTGGCCTGGTCAATA

4. Primers of RFLP for the mutant identification

RFLP	Forward primer	Reverse primer
topbp1 -RFLP	AGTTTTGACCCAAGCTGCAC	AAATAAGCATTTTTCTCAGCATCA

5. Primers for quantitative real-time PCR

Genes	Forward primer	Reverse primer
beta-actin	GATCTTCACTCCCCTTGTTCA	GGCAGCGATTTCCTCATC
c-myb	TGATGCTTCCCAACACAGAG	TTCAGAGGGAATCGTCTGCT
topbp1	CTTATTCAGCTCGCCAAACA	TAGCCGTCCGCACAAAGTA
p53	GTGGCTCTTGCTGGGACAT	GATGGCTGAGGCTGTTCTTC
p21	TGAGAACTTACTGGCAGCTTCA	AGCTGCATTCGTCTCGTAGC
mdm2	CAGGAGGAGGAGAAGCAGTG	AGGGAAAAGCTGTCCGACTT
cyclin G1	CATCTCTAAAAGAGGCTCTAGATGG	CACACAAACCAGGTCTCCAG

5. Primers for plasmid construction

Name	Forward primer	Reverse primer
topbp1 ^{WT}	AACGCTAGCCTGAGAGTTAATGGATGGCAAAGC	AACGCTAGCTCTCATACGGCTTTTCTTCAGTGT
topbp1 ^{cas003}	AACGCTAGCCTGAGAGTTAATGGATGGCAAAGC	AACGCTAGCCTGAGAGTTAATGGATGGCAAAGC
topbp1 probe	GCGGAATTCCATGTCACCATGTCGAAAGC	ATTGGTACCCCTGTGTAGGTGGCTCCATT
Topbp1^{R122E}	CTAACCTTGACAAAGAGGCAGAGAGTGAAGTGATGGATCTTA	TAAGATCCATCACTTCACTCTCTGCCTCTTTGTCAAGGTTAG
Topbp1^{R669E}	TGTCCAGGATTACTTTGTGGAGACGGCTAATCAGAGGAAG	CTTCCTCTGATTAGCCGTCTCCACAAAGTAATCCTGGACA
Topbp1^{W1156R}	AGAGTGAGCAGATCGTTAGGGATGACCCCAC	GTGGGGTCATCCCTAACGATCTGCTCACTCT
Topbp1$^{\Delta AAD}$	GAGATGAGGGAAAGTCTTCAAACAGCACGAGAAGAGA	TCTCTTCTCGTGCTGTTTGAAGACTTTCCCTCATCTC

6. topbp1 gRNA target site

Name	Sequence
topbp1 gRNA	5'-GGGAGCTACAGAGGGTCTGG-3'
